# p53 triggers mitochondrial apoptosis following DNA damage-dependent replication stress by the hepatotoxin methyleugenol

**DOI:** 10.1038/s41419-022-05446-9

**Published:** 2022-11-29

**Authors:** Max J. Carlsson, Anastasia S. Vollmer, Philipp Demuth, Daniel Heylmann, Diana Reich, Caroline Quarz, Birgit Rasenberger, Teodora Nikolova, Thomas G. Hofmann, Markus Christmann, Julia A. Fuhlbrueck, Simone Stegmüller, Elke Richling, Alexander T. Cartus, Jörg Fahrer

**Affiliations:** 1 Division of Food Chemistry and Toxicology, Department of Chemistry, Technical University of Kaiserslautern, 67663 Kaiserslautern, Germany; 2 Rudolf Buchheim Institute of Pharmacology, Justus Liebig University Giessen, 35392 Giessen, Germany; 3 Institute of Toxicology, University Medical Center, Johannes Gutenberg University Mainz, 55131 Mainz, Germany; 4 Present Address: Department of Dermatology, University Medical Center, 69120 Heidelberg, Germany

**Keywords:** Apoptosis, DNA, Liver cancer

## Abstract

Liver cancer is one of the most frequent tumor entities worldwide, which is causally linked to viral infection, fatty liver disease, life-style factors and food-borne carcinogens, particularly aflatoxins. Moreover, genotoxic plant toxins including phenylpropenes are suspected human liver carcinogens. The phenylpropene methyleugenol (ME) is a constituent of essential oils in many plants and occurs in herbal medicines, food, and cosmetics. Following its uptake, ME undergoes Cytochrome P450 (CYP) and sulfotransferase 1A1 (SULT1A1)-dependent metabolic activation, giving rise to DNA damage. However, little is known about the cellular response to the induced DNA adducts. Here, we made use of different SULT1A1-proficient cell models including primary hepatocytes that were treated with 1′-hydroxymethyleugenol (OH-ME) as main phase I metabolite. Firstly, mass spectrometry showed a concentration-dependent formation of *N*^2^-MIE-dG as major DNA adduct, strongly correlating with SULT1A1 expression as attested in cells with and without human SULT1A1. ME-derived DNA damage activated mainly the ATR-mediated DNA damage response as shown by phosphorylation of CHK1 and histone 2AX, followed by p53 accumulation and CHK2 phosphorylation. Consistent with these findings, the DNA adducts decreased replication speed and caused replication fork stalling. OH-ME treatment reduced viability particularly in cell lines with wild-type p53 and triggered apoptotic cell death, which was rescued by pan-caspase-inhibition. Further experiments demonstrated mitochondrial apoptosis as major cell death pathway. ME-derived DNA damage caused upregulation of the p53-responsive genes *NOXA* and *PUMA*, Bax activation, and cytochrome c release followed by caspase-9 and caspase-3 cleavage. We finally demonstrated the crucial role of p53 for OH-ME triggered cell death as evidenced by reduced pro-apoptotic gene expression, strongly attenuated Bax activation and cell death inhibition upon genetic knockdown or pharmacological inhibition of p53. Taken together, our study demonstrates for the first time that ME-derived DNA damage causes replication stress and triggers mitochondrial apoptosis via the p53-Bax pathway.

## Introduction

Liver cancer is one of the most frequent tumor entities worldwide with more than 800,000 new cases per year and highest rates in Eastern Asia as well as North Africa [1]. The predominant subtype represents hepatocellular carcinoma (HCC), which has been causally linked to chronic viral infection (hepatitis B or C), obesity, fatty liver disease, life-style factors such as alcohol consumption and smoking as well as food-borne carcinogens, in particular aflatoxins [2]. Moreover, genotoxic plant toxins including pyrrolizidine alkaloids and phenylpropenes are suspected human liver carcinogens [3, 4]. Hepatocellular carcinogenesis is a multistep process, which typically occurs on the basis of chronic liver inflammation with hepatocyte cell death and regenerative hyperproliferation. Chromosomal instability is a prevailing feature in HCC, which is already observed in chronically inflamed liver tissue and HCC precursor lesions [5, 6]. In the course of HCC development, a multitude of genetic and epigenetic alterations arises, thereby driving cancer progression [5, 6].

Methyleugenol (ME) belongs to the group of phenylpropenes, which comprises structurally related compounds such as safrole or estragole. ME is a secondary plant constituent and found in essential oils of different spices and herbs, with significant levels in basil, fennel, and nutmeg, but also fruits, e.g., in bananas and peaches [7, 8]. Due to its widespread occurrence in food, cosmetics, and herbal medicinal products, ME is taken up into the human body [9]. ME undergoes phase I metabolism catalyzed by cytochrome P450 (CYP) enzymes. The main product of this oxidative metabolism is 1′-hydroxymethyleugenol (OH-ME) [10]. CYP2E1 was found as most active enzyme catalyzing this reaction (1′-hydroxylation) in rat liver microsomes [11], whereas another study with human liver microsomes identified CYP1A2 as the most potent isoform [12]. OH-ME is then conjugated with a sulfo-group to yield 1′-sulfoxymethyleugenol, predominantly catalyzed via sulfotransferase 1A1 (SULT1A1) (Fig. S1). This intermediate is unstable and spontaneously decomposes under cleavage of sulfate into a highly reactive carbocation. The latter can attack the DNA, thereby forming *N*^6^-(trans-methylisoeugenol-3′-yl)-2′-deoxyadenosine (*N*^6^-MIE-dA) and *N*^2^-(trans-methylisoeugenol-3′-yl)-2′-deoxyguanosine (*N*^2^-MIE-dG) [10]. The importance of sulfotransferases (SULT) for the genotoxic effects of ME was demonstrated in different models, including *Salmonella typhimurium* and FVB/N mice, which were proficient or deficient for SULT1A [13–15]. It is further noteworthy that ME induced DNA adducts were found abundantly in human liver biopsies [16] as well as in human lung samples [17]. Both ME and its phase I metabolite OH-ME induced hepatic tumors in CD-1 mice [18]. A 2-year study performed in mice and rats revealed that ME is not only a hepatocarcinogen, but also causes tumor formation in other organs including glandular stomach, kidney, and mammary gland [19]. Hence, the International Agency for Research on Cancer (IARC) classified ME as possibly carcinogenic in humans (IARC group 2B) [20].

While the metabolism, genotoxicity, and carcinogenicity of ME are well described in literature, little is known about the interference of these adducts with DNA replication, the triggered DNA damage response (DDR), and downstream signaling pathways regulating the cellular response. In this study, we analyzed DNA damage induction upon exposure to the metabolite OH-ME in human liver cells and other cell models differing in their SULT1A1 and p53 status by using mass spectrometry, alkaline Comet assay and γH2AX immunofluorescence. The OH-ME triggered DDR was studied by western blot analysis and DNA replication was monitored by DNA fiber assay. Furthermore, cell death induction and the underlying mechanisms of apoptosis were investigated by viability assays, flow cytometry, western blot detection, quantitative PCR, and confocal microscopy. Finally, the role of p53 in ME-triggered cell death was detailed using pharmacological inhibitors, siRNA-mediated genetic knockdown, and the application of isogenic cell lines differing in their p53 status.

## Results

### The primary metabolite 1′-hydroxymethyleugenol (OH-ME) causes DNA damage in a SULT1A1-dependent manner

**Fig. 1:**
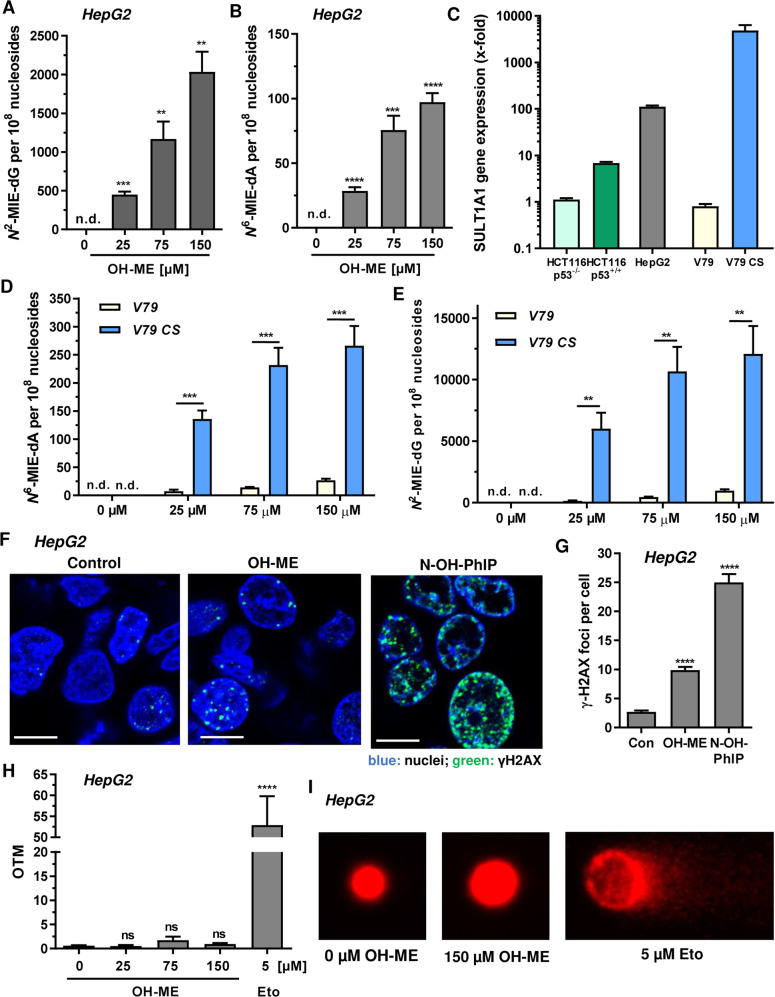
Impact of SULT1A1 on DNA damage induction by 1′-OH-methyleugenol in human liver cells and hamster fibroblasts. **A**, **B** Formation of *N*^2^-MIE-dG and *N*^6^-MIE-dA adducts in human HepG2 cells. Cells were exposed to increasing concentrations of OH-ME as indicated for 24 h. Isolated genomic DNA was digested to nucleosides and adduct levels were determined by UPLC-mass spectrometry using stable isotope dilution analysis (*n* = 4). **C** *SULT1A1* level in HepG2 cells and further cell lines used in this study. Gene expression was assessed by qPCR (*n* = 3). **D**, **E** Formation of *N*^2^-MIE-dG and *N*^6^-MIE-dA adducts in parental V79 cells and V79 cells proficient for human CYP1A2 and SULT1A (V79 CS). Cells were exposed to increasing concentrations of OH-ME as indicated for 24 h. Isolated genomic DNA was digested to nucleosides and adduct levels were measured by UPLC-mass spectrometry using stable isotope dilution analysis (*n* = 4). **F** Formation of the DNA damage marker γH2AX in HepG2 cells 24 h upon treatment with 150 µM OH-ME. DMSO was used as solvent control, while 50 µM N-OH-PhIP was included as positive control. Representative confocal microscopy images are shown (scale bar: 10 µm). **G** The number of γH2AX foci per nucleus were determined by ImageJ software (*n* = 3, 50–100 cells per experiment). **H** Analysis of DNA strand break induction by OH-ME. HepG2 cells challenged with increasing concentrations of OH-ME for 24 h were subjected to the alkaline Comet assay. Etoposide was included as positive control. OTM: olive tail moment (*n* = 3). **I** Representative images obtained by the Comet assay. Data in **A**–**H** are given as mean + SEM. Not significant, *p* > 0.05, **p < 0.01, ***p < 0.001, ****p < 0.0001. n.d. indicates not detected.

First, we investigated DNA adduct formation in human HepG2 liver cells, since the liver is the primary target organ of ME. HepG2 cells were challenged for 24 h with OH-ME, the main phase I metabolite of ME, and DNA adducts were analyzed by mass spectrometry. A clear concentration-dependent increase was observed for both DNA adducts, revealing *N*^2^-MIE-dG as main adduct and *N*^6^-MIE-dA as minor adduct (Fig. 1A, B). In order to detail the role of SULT1A1, we then made use of various cell models including HCT116 colorectal cancer cells proficient and deficient for p53 as well as V79 hamster fibroblasts and V79 cells genetically engineered for CYP1A2 and SULT1A1 expression (V79 CS). SULT1A1 expression in all cell models was then characterized on the gene and protein level. Gene expression analysis by qPCR showed that HepG2 liver cells express much higher SULT1A1 levels than HCT116-p53^+/+^ cells (Fig. 1C). Interestingly, p53-deficient HCT116 cells displayed the lowest expression level of all human cell lines. The highest SULT1A1 level was detected in V79 CS cells, whereas parental V79 cells displayed only very weak SULT1A1 expression (Fig. 1C). Comparable results were obtained by Western Blot analysis, with high SULT1A1 expression in HepG2 and V79 CS cells, moderate levels in HCT116-p53^+/+^ cells and very low levels in both parental V79 and HCT116-p53^−/−^ cells (Fig. S2A). Subsequently, V79 and V79 CS cells were exposed to OH-ME for 24 h and adduct levels were measured as described above. Both the *N*^2^-MIE-dG and the *N*^6^-MIE-dA adducts were generated in a concentration-dependent manner, reaching a plateau at the highest OH-ME concentration. This might be explained by saturation of the metabolic activation pathway at high substrate levels. Parental V79 cells showed much lower adduct levels than V79 CS cells, demonstrating the importance of SULT1A1 for the metabolic activation of OH-ME and the subsequent DNA adduct formation. In support of these findings, HCT116-p53^+/+^ cells with moderate SULT1A1 expression displayed lower adduct levels than HepG2 or V79 CS cells (Fig. S2B, C).

As another marker of DNA damage phosphorylation of histone 2AX, termed γH2AX, was analyzed by confocal immunofluorescence microscopy. γH2AX formation was observed in HepG2 cells exposed to OH-ME (Fig. 1F, G). The positive control N-OH-PhIP caused substantial γH2AX formation in HepG2 cells (Fig. 1F, G). N-OH-PhIP is a main phase I metabolite of the food-borne carcinogen 2-amino-1-methyl-6-phenylimidazo[4,5-b]pyridine (PhIP), which causes DNA damage upon metabolic activation by CYP1A2 and SULT1A1 [21]. A significant increase in γH2AX foci formation was detected in metabolically competent V79 CS cells 24 h after OH-ME treatment, whereas only background levels were observed in parental V79 cells (Fig. S3A, B). The positive control PhIP caused γH2AX foci only in V79 CS cells, but not in V79 cells (Fig. S3A, B). Similarly, an increased number of γH2AX foci was only detected in HCT116-p53^+/+^ cells incubated with increasing OH-ME concentrations, while no rise over γH2AX baseline levels was seen in HCT116-p53^−/−^ cells (Fig. S3C, D). This set of experiments further highlights the crucial role of SULT1A1 for OH-ME triggered DNA damage induction. Finally, DNA strand break formation was studied in HepG2 liver cells using an alkaline Comet assay. In contrast to the positive control etoposide, no significant increase of DNA strand breaks was detected 24 h after exposure to OH-ME (Fig. 1H, I). Lack of DNA strand break induction by OH-ME was also observed after 14 h of incubation, whereas etoposide clearly caused DNA strand breaks (Fig. S4A, B). Taken together, these findings demonstrate that OH-ME induces DNA adducts in a SULT1A1-dependent manner, with *N*^2^-MIE-dG as major lesion and *N*^6^-MIE-dA as minor lesion. This was associated with increased γH2AX levels, which indicates DDR activation, while DNA strand break induction was not detectable.

### OH-ME activates the DDR mainly via the ATR-CHK1-p53 axis

**Fig. 2:**
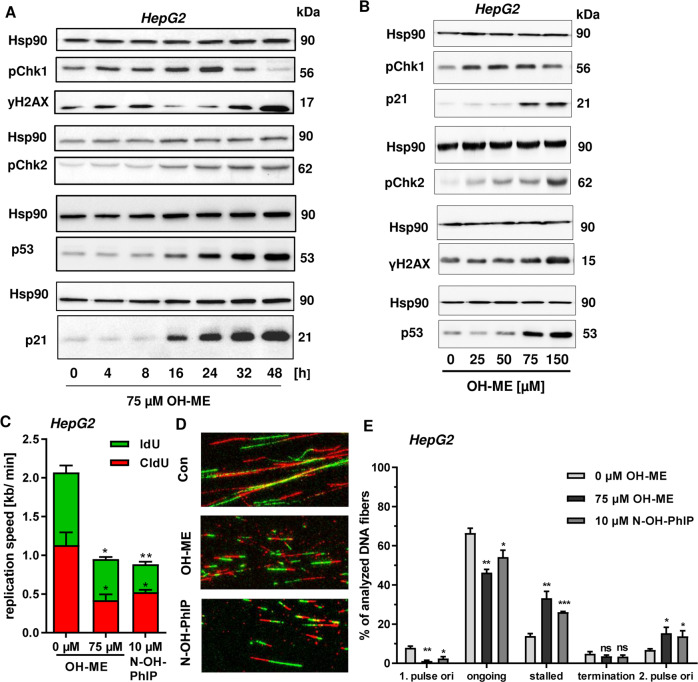
Activation of the DNA damage response by ME-derived DNA adducts and replication stress in human liver cells. **A** Time-dependent DNA damage response in HepG2 human liver cells. Cells were exposed to 75 µM OH-ME and incubated for up to 48 h. Samples were collected as indicated and subject to western blot analysis of pCHK1, γH2AX, pCHK2, p53, and p21. Hsp90 was detected as loading control. A representative blot is shown. **B** Concentration-dependent DNA damage response in HepG2 cells triggered by OH-ME. Cells were challenged with up to 150 µM OH-ME for 24 h. Subsequently, cell lysates were analyzed by SDS-PAGE and western blot detection as described in (**B**). A representative western blot is shown. **C** Impact of OH-ME on replication speed. HepG2 cells were treated for 24 h and replication speed was determined by DNA fiber assay using confocal microscopy. **D** Representative DNA fiber tracks of HepG2 cells exposed to OH-ME or N-OH-PhIP. **E** Distribution of replication structures assessed by the DNA fiber assay in HepG2 cells exposed to OH-ME or N-OH-PhIP for 24 h. Data in **C** and **E** are given as mean + SEM (*n* = 3). Not significant, *p* > 0.05, *p < 0.05, **p < 0.01. ***p < 0.001.

As a next step, DDR signaling upon OH-ME exposure was analyzed in more detail. A time-course experiment was performed in HepG2 cells, which were challenged with OH-ME for up to 48 h. CHK1 phosphorylation increased in a time-dependent manner and preceded γH2AX formation (Fig. 2A). CHK2 phosphorylation was also observed and increased at later time points. With similar kinetics, OH-ME caused accumulation of p53 and its downstream target p21 (Fig. 2A). In addition, the DDR activation by OH-ME was studied after 24 h in a concentration-dependent manner. Interestingly, CHK1 phosphorylation was detectable at 25 µM OH-ME and decreased at the highest concentration (Fig. 2B). In contrast to that, CHK2 phosphorylation was very strong at 150 µM OH-ME, coinciding with maximal levels of γH2AX, p53 and p21 (Fig. 2B). Importantly, OH-ME also triggered p53 accumulation and γH2AX formation in murine primary hepatocytes, which was corroborated by confocal immunofluorescence microscopy (Fig. S4C, D). To address the role of SULT1A1, parental V79 and V79 CS cells were treated with OH-ME for up to 24 h. Western blot analysis revealed phosphorylation of CHK1 only in V79 CS cells, which was clearly detectable 8 h after treatment with OH-ME (Fig. S4E). Similarly, γH2AX levels increased after OH-ME treatment and culminated after 24 h. Moreover, CHK1 phosphorylation and γH2AX formation occurred in a concentration-dependent manner in V79 CS cells (Fig. S4F). In addition to that, HCT116-p53^+/+^ and HCT116-p53^−/−^ cells were exposed to OH-ME for 24 h. Similar to the findings in HepG2 (Fig. 2B), OH-ME treatment resulted in γH2AX formation and accumulation of pCHK1 as well as p53 in HCT116-p53^+/+^ cells. In contrast to that, no effects on the DDR were observed in p53-deficient HCT116 cells (Fig. S5A–D). Taken together, these findings illustrate the causal link between SULT1A1 expression, DNA adduct formation and subsequent DDR activation. Since ATR-mediated CHK1 phosphorylation is indicative of replication stress, we tested whether OH-ME induced DNA adducts interfere with the DNA replication. By means of a DNA fiber assay, OH-ME was shown to significantly reduce the replication speed in HepG2 cells, which was also observed for the positive control N-OH-PhIP (Fig. 2C). Further analysis of DNA fiber tracks revealed an increased number of stalled replication forks and a concomitant decrease in ongoing replication forks after incubation with OH-ME or the positive control N-OH-PhIP (Fig. 2D, E). Similar results were obtained in HCT116 cells exposed to OH-ME (Fig. S5E–G). In conclusion, the data provide evidence that OH-ME induced DNA adducts trigger the DDR mainly via the ATR-CHK1-p53 signaling cascade, indicative of replication stress. In line with this observation, the formed DNA adducts caused replication fork stalling and decreased the replication speed.

### OH-ME triggered DNA damage impairs cell viability and causes cell death

**Fig. 3:**
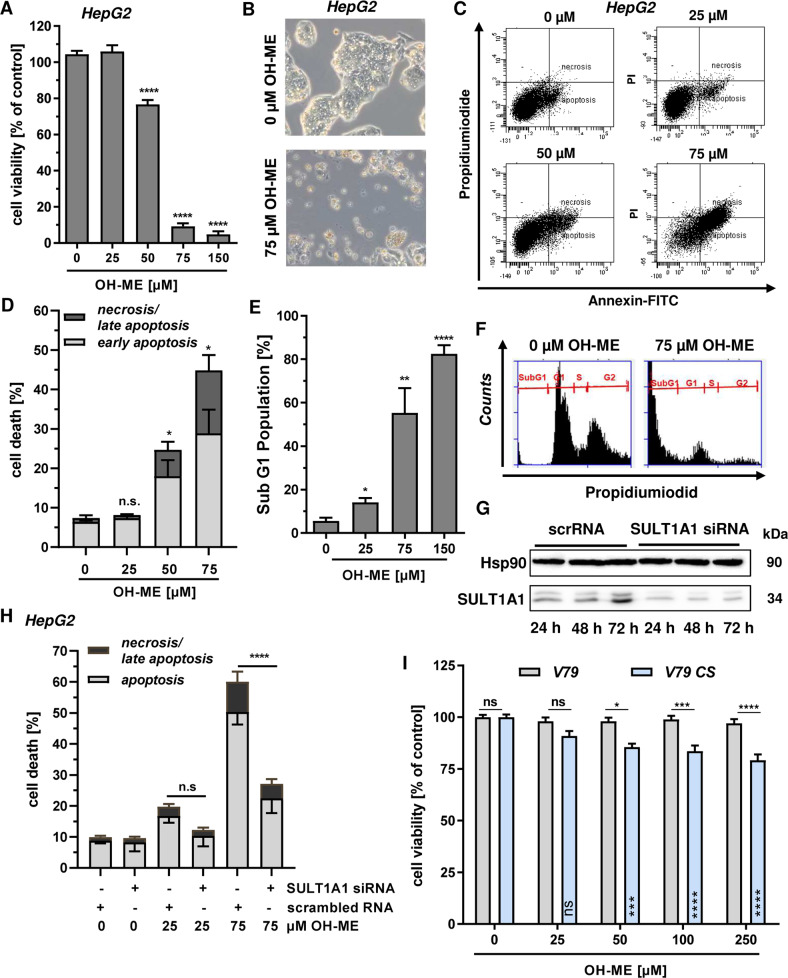
Cytotoxicity of ME-triggered DNA damage and impact of SULT1A1. **A** Viability of HepG2 cells challenged with increasing concentrations of OH-ME for 72 h. Viability was determined by the MTS assay (*n* = 3). **B** Morphology of HepG2 cells treated with 75 µM OH-ME or solvent control (0 µM) as revealed by light microscopy. **C**, **D** Cell death induction by OH-ME in HepG2 cells. Cells were exposed to OH-ME for 72 h followed by Annexin V-FITC/PI staining and analysis by flow cytometry (*n* = 3). Representative dot plots of Annexin V-FITC/PI staining are shown. **E**, **F** Analysis of subG1 population in HepG2 cells exposed to increasing concentrations of OH-ME for 72 h. Cells were harvested, stained and subG1 population indicative of apoptosis was determined by flow cytometry (*n* = 4). Representative histograms are shown. **G** siRNA-mediated knockdown of SULT1A1 in HepG2. Cells were transfected with SULT1A1 siRNA or scrambled siRNA (scrRNA) and SULT1A1 expression was monitored for up to 72 h by western blot analysis. Hsp90 served as loading control. **H** Impact of SULT1A1 knockdown on OH-ME triggered cell death. HepG2 cells were transfected as described above and then challenged with OH-ME. Cell death induction was measured by Annexin V-FITC/PI staining after 72 h (*n* = 3). **I** Viability of V79 and V79 CS cells upon treatment with OH-ME. Cells were exposed to increasing OH-ME concentrations (0–250 µM) and viability was assessed after 72 h using the MTS assay (*n* = 3). Data in **A**–**I** are shown as mean + SEM. Ns: not significant, *p* > 0.05, *p < 0.05, **p < 0.01, ***p < 0.001, ****p < 0.0001.

In our next experiments, we addressed the question whether the ME-derived DNA adducts trigger cytotoxicity. HepG2 liver cells were incubated with OH-ME for 72 h and viability was assessed by MTS assay. We observed a concentration-dependent cytotoxicity with a drop in viability below 20% at a concentration of 75 µM OH-ME (Fig. 3A). The high cytotoxicity was also visible by phase contrast microscopy, which revealed a decreased cell density as well as many round and detached cells following OH-ME treatment (Fig. 3B). In order to detail the mode of cytotoxicity, HepG2 cells were exposed to OH-ME for 72 h and Annexin V-FITC/PI staining was performed. Flow cytometry showed a concentration-dependent induction of both early apoptosis and late apoptosis/necrosis after OH-ME treatment, with significantly elevated cell death levels at 50 µM OH-ME (Fig. 3C, D). This result was checked by subG1 analysis, which is indicative of apoptosis and subsequent DNA fragmentation. In line with the previous finding, OH-ME significantly increased the subG1 population depending on its concentration (Fig. 3E, F). To analyze the requirement of SULT1A1-dependent metabolic activation for the observed cytotoxicity, SULT1A1 expression was transiently downregulated in HepG2 cells using siRNA. Western blot analysis confirmed efficient SULT1A1 knockdown over a period of 72 h (Fig. 3G). Subsequently, cell death induction upon OH-ME treatment was analyzed by Annexin V-FITC/PI staining and flow cytometry, revealing a strong protection of the cells following SULT1A1 knockdown (Fig. 3H and Fig. S6A). This was also mirrored in the observations by phase contrast microscopy (Fig. S6B). Furthermore, V79 and V79 CS cells were incubated with OH-ME for 72 h and viability was assessed using the MTS assay. OH-ME did not affect V79 cells at all, but reduced the viability of metabolically competent V79 CS cells in a concentration-dependent manner (Fig. 3I). Despite high SULT1A1 expression and higher DNA adduct levels, the cytotoxic effects in V79 CS cells were rather moderate in comparison to those in HepG2 cells. This is likely attributable to the expression of mutated and non-functional p53 in V79 cells [22], pointing to a central role of p53 in OH-ME triggered cell death (see also below). Altogether, our findings show that OH-ME is cytotoxic at higher concentrations and induces apoptosis. The cellular sensitivity towards OH-ME was clearly dependent on SULT1A1 expression and subsequent DNA adduct formation, with the strongest cytotoxicity in HepG2 cells that express wild-type p53.

### OH-ME induced DNA damage results in caspase-dependent mitochondrial cell death

**Fig. 4:**
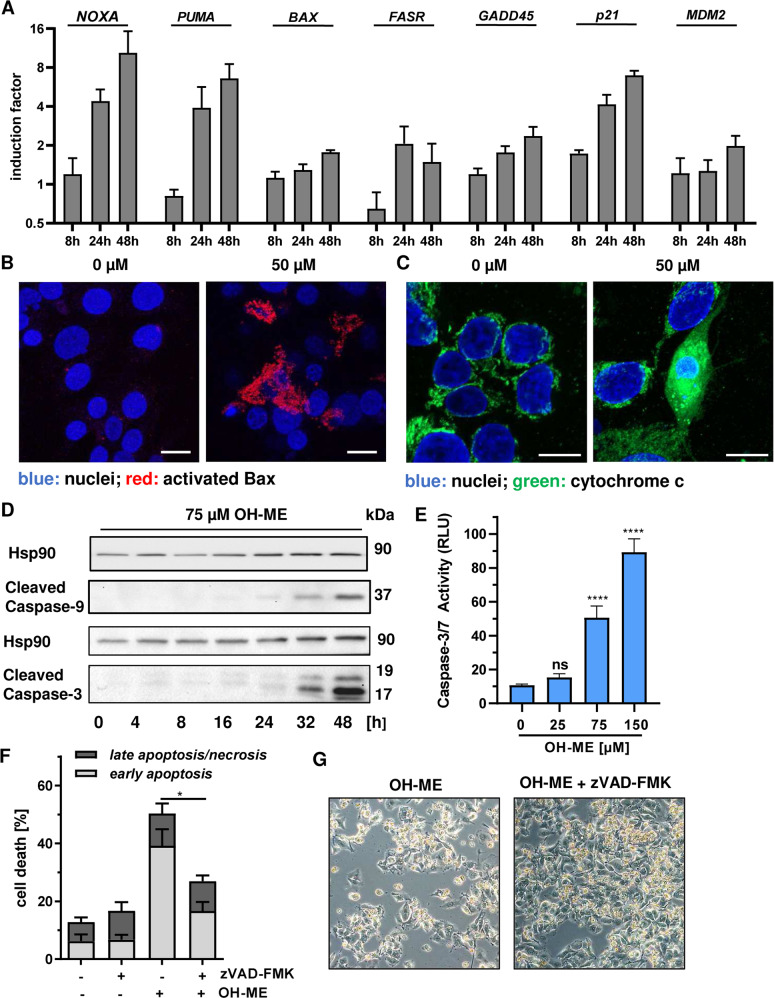
ME-derived DNA damage triggers caspase-dependent mitochondrial apoptosis. **A** Time-dependent expression of pro-apoptotic genes (*NOXA, PUMA, BAX, FASR*) and other p53-related genes (*GADD45, p21, MDM2*) in HepG2 cells after treatment with 75 µM OH-ME for up 48 h. Gene expression was assessed by qPCR (*n* = 4). **B** Assessment of Bax activation in HepG2 cells treated with 50 µM OH-ME for 48 h. Cells were fixed, processed and immunostained for activated Bax (red), while nuclei were visualized by DAPI. Images were acquired by confocal microscopy. Scale bar: 20 µm. **C** Analysis of cytochrome c release in HepG2 cells after OH-ME treatment by confocal microscopy. Cells were incubated for 48 h, fixed and stained for cytochrome c (green). Nuclei were counterstained with DAPI (blue). Samples were then analyzed by confocal microscopy and processed by ZEN software. Scale bar: 20 µm. **D** Time-dependent caspase cleavage in HepG2 following OH-ME treatment. Cells were incubated with 75 µM OH-ME for up to 48 h. Samples were analyzed by SDS-PAGE and western blot detection for caspase-9 and caspase-3 cleavage. Hsp90 served as loading control. A representative blot is shown. **E** Determination of caspase-3/-7 activity upon OH-ME exposure. Cells were treated for 24 h with increasing concentrations of OH-ME, harvested and analyzed for caspase-3/-7 activity using a luminogenic substrate (*n* = 3). **F** Impact of caspase inhibition on OH-ME triggered cell death. HepG2 cells were treated with 75 µM OH-ME in the absence or presence of the pan-caspase inhibitor zVAD-FMK for 48 h. Cell death induction was analyzed by Annexin V-FITC/PI staining and flow cytometry (*n* = 3). **G** Representative microscopy images of HepG2 cells treated with OH-ME in the absence or presence of the pan-caspase inhibitor zVAD-FMK. Data in **A**, **E**, and **F** are given as mean + SEM. Ns: not significant, *p* > 0.05, *p < 0.05, ****p < 0.0001.

To characterize the mode of cell death triggered by OH-ME in HepG2 cells, the time-dependent expression of various apoptosis- and p53-related genes were determined by Real-Time qPCR. To this end, cells were treated with either 75 µM OH-ME or the solvent control and then incubated for up to 48 h. qPCR analysis showed the time-dependent upregulation of well-known p53 targets, such as *p21* and *GADD45* that are involved in cell cycle arrest following DNA damage, as well as its negative regulator *MDM2* (Fig. 4A right panel). Furthermore, the expression of several pro-apoptotic genes was induced by OH-ME, particularly the p53-regulated genes *PUMA* and *NOXA* (Fig. 4A, left panel). In turn, anti-apoptotic genes were either unaffected (*Survivin, BCL2, c-IAP1*) or were downregulated as in the case of *c-IAP2* (Fig. S7A, B). Since both *PUMA* and *NOXA* encode for BH3-only proteins involved in the activation of Bax, confocal immunofluorescence microscopy was performed in HepG2 cells. In line with our hypotheses, a strong Bax activation was observed after treatment with 50 µM OH-ME (Fig. 4B and Fig. S7C). This was accompanied by release of cytochrome c into the cytoplasm and partial nuclear translocation as evidenced by confocal microscopy, while solvent treated control cells show mitochondrial localization of cytochrome c (Fig. 4C). Consistent with the intrinsic apoptosis pathway, western blot analysis showed cleavage of the initiator caspase-9 after OH-ME treatment, whereas caspase-8 cleavage involved in the extrinsic apoptosis pathway was not observed (Fig. S7D). Finally, OH-ME caused cleavage and activation of the executioner caspase-3, which was demonstrated by western blot detection and a luminescent caspase activity assay (Fig. 4D, E). In order to corroborate that caspase activation is required for OH-ME-dependent cell death, the pan-caspase inhibitor zVAD-FMK was used. Intriguingly, pan-caspase inhibition significantly reduced cell death as shown by Annexin V-FITC/PI staining (Fig. 4F) and phase-contrast microscopy (Fig. 4G).

In summary, our data clearly show that OH-ME causes an upregulation of p53-regulated pro-apoptotic genes, which promote Bax activation and release of cytochrome c from mitochondria, finally resulting in caspase-dependent mitochondrial cell death.

### The tumor suppressor p53 is crucial for OH-ME-triggered mitochondrial apoptosis via Bax activation

**Fig. 5:**
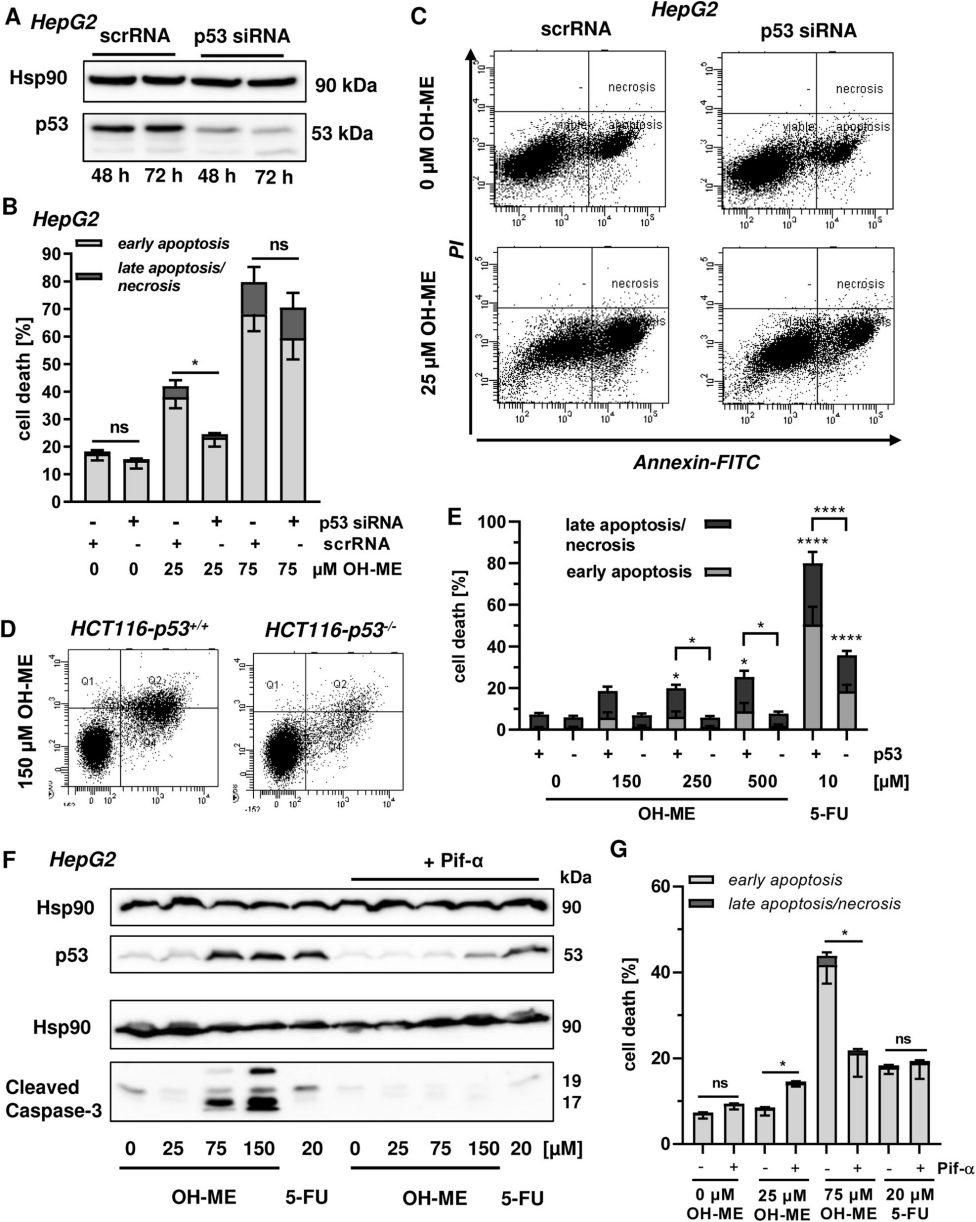
Role of p53 in OH-ME triggered apoptosis. **A** Transient knockdown of p53 in HepG2 cells. Cells were transfected with siRNA directed against p53 (p53 siRNA) or scrambled siRNA (scrRNA) and harvested after 48 or 72 h. p53 expression was then analyzed by SDS-PAGE and western blot detection. Hsp90 served as loading control. **B** Knockdown of p53 and cell death induction by OH-ME. HepG2 cells were transfected with p53 siRNA or scrambled RNA followed by treatment with OH-ME. Cell death induction was analyzed by Annexin V-FITC/PI staining and flow cytometry (*n* = 4). **C** Representative dot plots of Annexin V-FITC/PI staining. **D**, **E** Isogenic HCT116 cells proficient or deficient for p53 were challenged with increasing OH-ME concentrations for 72 h. 5-FU served as positive control. Cell death was assessed as described above (*n* = 4). Representative dot blots are depicted. **F** Pharmacological inhibition of p53 and impact on caspase-3 cleavage triggered by OH-ME. HepG2 cells were pre-treated or not with the p53 inhibitor pifithrin-α for 2 h and then challenged with increasing concentrations of OH-ME (0–150 µM) for 24 h. Samples were subjected to SDS-PAGE followed by western blot analysis of p53 and cleaved caspase-3. As loading control, Hsp90 was detected. **G** Pharmacological inhibition of p53 and cell death induction by OH-ME. HepG2 cells were pre-incubated with pifithrin-α or not and then exposed to OH-ME for 48 h. Cell death was assessed by Annexin V-FITC/PI staining and flow cytometry. Data in **B**, **E**, and **G** are given as mean + SEM. Ns: not significant, *p* > 0.05, *p < 0.05.

**Fig. 6:**
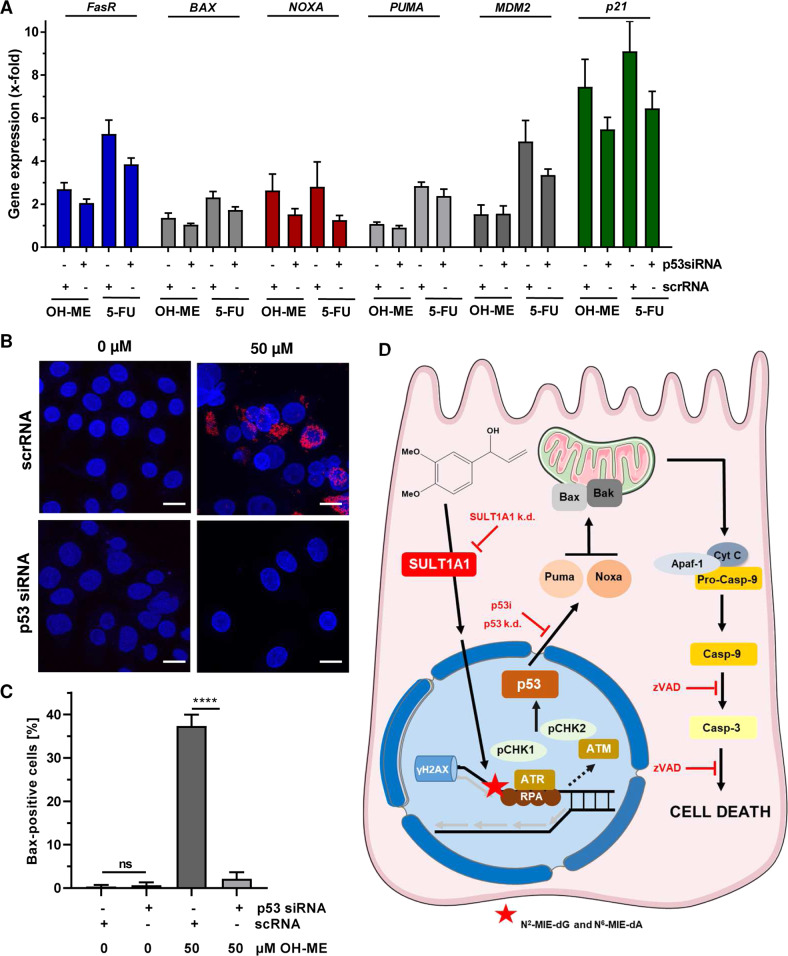
Impact of p53 on pro-apoptotic gene expression and Bax activation upon ME-derived DNA damage. **A** Expression of pro-apoptotic genes (*NOXA, PUMA, BAX, FASR*) and other p53-related genes (*p21, MDM2*) in HepG2 cells after p53 knockdown and treatment with 75 µM OH-ME for 24 h. Gene expression was assessed by qPCR (*n* = 4). Data are expressed as mean + SEM. **B** Assessment of Bax activation in HepG2 cells following p53 knockdown and treatment with 50 µM OH-ME for 48 h. Cells were fixed, processed, and immunostained for activated Bax (red), while nuclei were visualized by DAPI. Images were acquired by confocal microscopy. Scale bar: 20 µm. **C** Quantitative evaluation of Bax-positive cells (*n* = 3). Data are shown as mean + SEM. Ns: not significant, ****p > 0.0001. **D** Model of ME-triggered replication stress, DNA damage response, and cell death induction via p53 in liver cells. ME causes *N*^2^-MIE-dG and *N*^6^-MIE-dA adducts via its primary metabolite OH-ME, which is activated by sulfate conjugation catalyzed by SULT1A1. Genetic knockdown of SULT1A1 or low intrinsic SULT1A1 levels strongly attenuate DNA adduct formation, highlighting its critical role. The induced DNA adducts cause replication stress as evidenced by γH2AX formation and CHK1 phosphorylation. ATR-CHK1 activation together with delayed CHK2 activation, presumably catalyzed by ATM, lead to p53 stabilization. The tumor suppressor protein then triggers a cell death program via upregulation of the BH3-only factors *PUMA* and *NOXA*, which result in Bax activation and cytochrome c release from mitochondria. This causes apoptotic cell death via caspase-9 and caspase-3 cleavage, which is rescued by the pan-caspase inhibitor zVAD. Importantly, this cell death cascade is driven by p53 as demonstrated by pharmacological p53 inhibition or its genetic ablation. This figure was created using Servier Medical Art templates, which are licensed under a Creative Commons Attribution 3.0 Unported License; https://smart.servier.com.

As p53 accumulation and transcriptional upregulation of p53 target genes were observed, we wished to study the role of p53 in more detail using pharmacological and genetic approaches. First, HepG2 cells were transfected with siRNA against p53 or scrambled control RNA and p53 expression was monitored by western blot analysis, revealing an efficient p53 knockdown for up to 72 h (Fig. 5A). Importantly, p53 levels remained at baseline levels even after challenge with up to 150 µM OH-ME for 24 h (Fig. S8A). Using these settings, cell death induction was analyzed by Annexin V-FITC/PI staining and flow cytometry. The siRNA-mediated p53 knockdown conferred resistance to the cytotoxicity triggered by OH-ME as indicated by significantly lower levels of cell death induction (Fig. 5B, C). It should be mentioned that we observed a higher sensitivity of transfected cells towards OH-ME, generally increasing the cell death rates already at 25 µM OH-ME. The contribution of p53 was further analyzed in HCT116-p53^+/+^ and HCT116-p53^−/−^ cells that were challenged with OH-ME for 72 h. In p53-wild-type HCT116 cells, a concentration-dependent increase in cell death was detected, while HCT116 cells with a *p53* knockout were not affected at all (Fig. 5D, E, Fig. S8B). These results were confirmed by phase contrast microscopy (Fig. S8C). It should be mentioned here that HCT116-p53^−/−^ cells also have lower SULT1A1 levels (see above), thereby dampening the formation of OH-ME-derived DNA adducts. The anticancer drug 5-FU showed a differential cell death response in HCT116 cells depending on their p53 status (Fig. 5E), as reported previously [23]. Furthermore, the established small molecule inhibitor pifithrin-α was used to block p53 activation. To this end, cells were pre-treated or not with pifithrin-α followed by incubation with increasing concentrations of OH-ME for 24 h. Western blot analysis revealed almost complete inhibition of p53 accumulation and the concomitant caspase-3 cleavage in the presence of pifithrin-α, whereas without inhibitor pre-treatment OH-ME caused p53 stabilization and strong caspase-3 cleavage at high OH-ME concentrations (Fig. 5F). In agreement with these findings, cell death induction by OH-ME was strongly reduced upon p53 inhibitor treatment (Fig. 5G). The genotoxic anticancer drug 5-FU was included as positive control and caused p53 accumulation as well as moderate cell death levels. These effects were hardly blocked by pifithrin-α treatment, which is in contrast to the observations for OH-ME (Fig. 5F, G). The cytoprotective effect of p53 inhibition by pifithrin-α was confirmed in a cell viability assay (Fig. S8D). As a next step, we analyzed the contribution of p53 to apoptotic gene expression and Bax activation in HepG2 cells. Cells were transfected with p53 siRNA or scrambled RNA under the conditions described before (Fig. 5A) and treated with 75 µM OH-ME for 24 h to assess early changes in gene expression. qPCR analysis showed a reduced expression of the pro-apoptotic genes *FasR*, *BAX,* and *NOXA* as well as the p53 downstream target p21 following p53 knockdown, while the effects on *PUMA* induction were generally weak in this experimental setup (Fig. 6A). A similar pattern was observed after treatment with the positive control 5-FU (Fig. 6A). Finally, we assessed Bax activation in HepG2 cells with and without p53 knockdown after exposure to 50 µM OH-ME for 48 h. Please note that higher OH-ME concentrations result in massive loss of cells on the cover slips during the immunofluorescence procedure. Knockdown of p53 dramatically reduced the number of cells with activated Bax, as determined by confocal microscopy (Fig. 6B, C). Altogether, our results demonstrate that p53 activation by ME-derived DNA adducts triggers mitochondrial apoptotic cell death via Bax activation, which can be rescued by genetic knockdown or pharmacological inhibition of p53. Figure 6D illustrates the pathways involved in this process and highlights the key roles of both SULT1A1 and p53.

## Discussion

Using several cell models, we demonstrated that *N*^2^-MIE-dG is the major DNA adduct upon OH-ME exposure and its formation clearly depends on the SULT1A1 expression level. Cells with elevated SULT1A1 level (HepG2 and V79 CS) display higher DNA adduct levels as compared to cells with moderate SULT1A1 expression (HCT116-p53^+/+^). An interesting observation are the different SULT1A1 levels found in HCT116-p53^+/+^ and HCT116-p53^−/−^ cells, which is in line with a previous study [24]. In parental V79 cells with very low endogenous SULT1A1 expression, DNA adduct formation was strongly reduced. This is in line with previous studies using *Salmonella typhimurium* genetically engineered for SULT1A1 expression and transgenic mouse models differing in their SULT1A1 status [14, 15]. *N*^2^-MIE-dG adducts were also found abundantly in human liver biopsies and positively correlated with the expression level of SULT1A1 [16, 25], emphasizing its crucial role for metabolic activation and subsequent DNA adduct formation. We then showed the formation of γH2AX foci, an established DNA damage marker for DNA double-strand breaks and replication stress, in cells exposed to OH-ME, including primary murine hepatocytes. Noteworthy, the γH2AX levels were not as high as in cells exposed to the heterocyclic aromatic amine PhIP, which causes bulky DNA adducts [21]. Moderately increased γH2AX levels were also observed in HT29 CRC cells following exposure to OH-ME and other minor metabolites of ME, such as 3′-oxomethylisoeugenol [26]. Despite DNA adduct and γH2AX formation, no DNA strand breaks were monitored by the alkaline Comet assay. This could be attributable to the fact that DNA adducts undergoing base excision repair (BER) or nucleotide excision repair (NER) are hardly detectable by the Comet assay [27]. The DNA single-strand break repair intermediates generated during BER and NER are transient in nature and quickly sealed. Our findings are consistent with the absence of DNA strand break formation in liver tissue of rats exposed to increasing doses of ME as measured by the Comet assay, although significant DNA adduct formation was demonstrated at these dose levels [28].

Further analysis of the DDR triggered by ME-derived DNA adducts revealed phosphorylation of both CHK1 and CHK2 in a time- and concentration-dependent manner. Interestingly, CHK1 phosphorylation at serine 345, which is known to be catalyzed by ATR [29], preceded γH2AX formation and occurred early after OH-ME exposure. In turn, CHK2 phosphorylation at threonine 68, which is known to be catalyzed by ATM [30], was found particularly after treatment with high OH-ME concentrations and after prolonged incubation periods. These findings are supported by the observation that OH-ME causes moderate activation of the apical DDR kinases ATM and ATR in HT29 CRC cells [31]. Our data also showed an accumulation of p53 and its downstream target p21 in liver cells, strongly suggesting that MIE DNA adducts trigger an ATR-CHK1-p53 driven DDR as replication stress response. This was substantiated by DNA fiber assays, showing that OH-ME treatment decreases DNA replication speed and causes replication fork stalling. These effects were not as pronounced as following PhIP treatment, which forms bulky DNA adducts [32]. The C8-dG-PhIP adduct is known to distort the DNA helix and to block replicative DNA polymerases [21, 33]. Both the *N*^2^-MIE-dG and *N*^6^-MIE-dA adducts are less bulky and likely more flexible due to the propenyl-linker between the modified DNA base and the dimethoxyphenyl moiety. It is therefore conceivable that these DNA lesions may have a lower potential to stall replicative DNA polymerases or might be bypassed efficiently by DNA translesion synthesis. *N*^2^-dG adducts of benzo[*a*]pyrene were shown to block replicative polymerases in vitro, which was recovered by the bacterial Y-family polymerase DNA Pol IV [34]. Molecular modeling studies using DNA Pol IV with the two main PhIP-induced DNA adducts, i.e., C8-PhIP-dG and *N*^2^-PhIP-dG, suggest less steric hindrance for the minor groove *N*^2^-dG DNA adduct [35]. Bearing in mind that C8-PhIP-dG is the major PhIP adduct and *N*^2^-MIE-dG the major ME adduct, this could explain the different potencies in replication fork stalling and DDR activation.

Replication stress can have detrimental consequences for the cell, including genomic instability and cell death [36]. Indeed, exposure of cells to OH-ME concentrations above 25 µM caused cytotoxicity, which was most pronounced in HepG2 cells that express wt-p53 and display high SULT1A1 activity [37, 38]. In contrast to that, V79 CS were less sensitive despite high SULT1A1 levels. As mentioned before, this is likely attributable to the expression of mutated and non-functional p53 in V79 cells [22]. Moreover, moderate DNA adduct levels in wild-type HCT116 cells with low SULT1A1 expression after OH-ME treatment resulted in significant cytotoxicity, highlighting the importance of functional p53. Cytotoxicity was also reported in primary rat hepatocytes exposed to either ME or its primary metabolite OH-ME [10]. In this regard, it should be mentioned that p53 accumulation in murine primary hepatocytes was observed within our study. More detailed cell death analysis revealed for the first time that OH-ME induces mitochondrial apoptosis in liver cells, which was triggered by the upregulation of the pro-apoptotic BH3-only factors *PUMA* and *NOXA*, leading to Bax activation, mitochondrial outer membrane permeabilization (MOMP) and subsequent release of cytochrome c. This provoked caspase-9 and caspase-3 activation, finally resulting in DNA fragmentation. Importantly, pan-caspase inhibition was able to rescue the cells from apoptosis induction. The requirement of p53 for OH-ME triggered apoptosis in liver cells was demonstrated by pharmacological abrogation with pifithrin-α and genetic knockdown of p53, which repressed pro-apoptotic gene expression, Bax activation, caspase 3 cleavage, and apoptosis. The observation that p53 knockdown in HepG2 cells almost completely blocks Bax activation, although the pro-apoptotic gene expression was not fully blunted, is very likely explained by the transcription-independent mechanisms of p53-triggered apoptosis. Cytoplasmic p53 was reported to directly bind to and activate Bax and Bak, thereby promoting MOMP [39]. Interestingly, HT29 cells with mutant p53 showed only moderate levels of caspase-3 activity despite exposure to high OH-ME concentrations [31]. Together with our results obtained in V79 CS cells as well as the pharmacological and genetic approaches in HepG2 liver cells, this highlights the crucial role of p53 in apoptosis induction upon OH-ME exposure. Cells with a high burden of MIE adducts are thus eliminated by apoptosis, preventing the generation of initiated cells with mutations that would promote liver carcinogenesis. Intriguingly, sporadic p53 mutations are found in more than 30% of all human liver cancers [39], underlining the important role of p53 in tumor suppression.

It is well established that ME and its primary metabolite OH-ME cause liver cancer in rodent models [19]. An early event in this process are mutations in the proto-oncogene *ß-catenin*, which were found in liver tumors of mice exposed to increasing doses of ME [40]. These were mostly point mutations in four *ß-catenin* codons, resulting in ß-catenin protein accumulation [40]. Another study described a dose-dependent increase in the mutational burden of hepatocellular carcinomas in mice treated with ME [41]. The ME induced liver tumors clustered closely to the Catalogue of Somatic Mutations in Cancer (COSMIC) signatures 4 and 24 as shown by exome sequencing [41]. Interestingly, these signatures have been previously identified in human hepatocellular carcinoma with known exposure to aflatoxins and benzo[a]pyrene [42], which might indicate common mechanism of mutagenesis. ME was classified as possibly carcinogenic to humans (IARC group 2B) [20]. The human relevance is reflected by the finding that formation of DNA adducts already occurred after a single administration of ME (50 µg/kg body weight) to mice with human SULT1A expression at a realistic dietary exposure scenario [9, 15]. In support of these data, *N*^2^-dG-MIE and, to a lesser extent, *N*^6^-MIE-dA adducts were detected in human liver samples and in human lung tissue, correlating with *SULT1A1* copy number [15, 17, 25]. Our study provided evidence that, despite harboring significant levels of DNA adducts, liver cells are not eliminated via apoptotic cell death at concentrations of 25 µM OH-ME and below. These findings suggest that upon ME exposure liver tumor initiation might be facilitated due to the attenuated toxicity of the formed DNA lesions and survival of damaged cells. Another important aspect is the repair of ME-induced DNA adducts, which would counteract the subsequent fixation of mutations. A recent study with the structurally related phenylpropene estragole in primary rat hepatocytes revealed a certain persistence of *N*^2^-IE-dG adducts over 48 h, pointing to a delayed or inefficient repair [43]. Consistent with this notion, repeated pulse treatment of liver cells with estragole resulted in an accumulation of the respective *N*^2^-IE-dG adduct [43]. Therefore, more studies are required to identify the pathways involved in the removal of DNA adducts induced by phenylpropenes in liver cells.

Taken together, our study demonstrates for the first time that ME-derived DNA adducts cause DNA replication stress with concomitant activation of the ATR-CHK1-p53 axis and γH2AX formation. While moderate DNA adduct levels were tolerated well, higher levels induced caspase-dependent mitochondrial apoptosis in liver cells via the p53-Bax pathway, thereby limiting the survival of cells prone to acquire mutations.

## Materials and methods

### Cell culture

V79 Chinese hamster cells and V79-derived cells stably expressing both human cytochrome P450 1A2 (CYP1A2) and human sulfotransferase 1A1 (SULT1A1) [44], designated V79 CS, were provided by Dr. Hansruedi Glatt (German Institute of Human Nutrition, Potsdam-Rehbrücke, Germany) in 2014. V79 and V79 CS were authenticated by their fibroblast-like morphology and differential response to PhIP [21]. Cells were maintained in DMEM-Ham’s F12 medium supplemented with 5% FCS, 100 U/mL penicillin and 100 µg/mL streptomycin. Wild type (WT) HCT116 colorectal cancer cells as well as their isogenic counterpart HCT116-p53^−/−^ were provided by Dr. Bert Vogelstein (John Hopkins University, Baltimore, USA) in 2012. Cells were re-authenticated by p53 immunoblotting and by their characteristic differential response to 5-FU [23]. HCT116 cells were maintained in DMEM supplemented with 10% FCS and antibiotics (100 U/mL penicillin and 100 μg/mL streptomycin). HepG2 cells that express wild-type p53 [45] were obtained from DSMZ (Braunschweig, Germany) in 2016 and re-authenticated by their p53 status and typical cell morphology. HepG2 cells were maintained in DMEM + 10% FCS and antibiotics. All cell lines were cultured at 37 °C in a humidified atmosphere of 5% CO_2_ and 95% air. Cell culture medium and antibiotics were from Gibco Life Technologies (Darmstadt, Germany), while FCS was obtained from PAN-Biotech (Aidenbach, Germany). Cell lines were mycoplasma negative as routinely demonstrated by PCR using the Venor®GeM Classic kit (Minerva Biolabs, Berlin, Germany) and immunofluorescence microscopy with nuclear staining. All cell lines were characterized for SULT1A1 expression on the gene and protein level as described (Fig. 1C and Fig. S2A).

### Isolation of primary murine hepatocytes

Male C57BL6/J mice (typical age: 8–12 weeks) obtained from our in-house animal facility were anesthetized by i.p. administration of pentobarbital. Primary murine hepatocytes (pMH) were isolated by an adjusted two-step EGTA/collagenase-perfusion as described previously [46]. Cells were only used for further experiments if cell viability, determined by trypan blue exclusion, exceeded 85%. pMH were seeded on collagen-coated cell culture dishes or plates in DMEM supplemented with 10% FCS and 1% Pen/Strep and allowed to attach for 3 to 4 h. Unattached cells were removed by medium exchange and cells were treated with increasing concentrations of OH-ME as indicated. DMSO was included as solvent control (final concentration 0.1%).

### Compounds and treatment

1′-Hydroxymethyleugenol (OH-ME) was synthesized as reported previously [10] with a purity of approximately 99 % determined by ^1^H-NMR. A stock solution with a final concentration of 250 mM was prepared in DMSO. PhIP and N-OH-PhIP were kindly provided by Dr. Albrecht Seidel (Biochemical Institute of Environmental Carcinogens, Großhansdorf, Germany). Both compounds were dissolved in DMSO at a stock concentration of 30 mM and 10 mM, respectively. The pan-caspase inhibitor z-VAD-FMK (20 mM in DMSO) was from Selleck Chemicals (Houston, USA). The p53 inhibitor pifithrin-α (100 mM in DMSO, final concentration: 20 µM) was purchased at Hycultec (Beutelsbach, Germany). Cells were treated as indicated with the compounds and inhibitors.

### Isolation of genomic DNA and digestion

Isolation of genomic DNA was performed by standard chloroform/phenol-extraction as published [47]. To this end, pelleted cells were suspended in 800 µL of lysis buffer (35 mM TRIS, 0.56 mM NaEDTA, 0.018 mM acetic acid, 1% SDS and 0.05% triton-X-100 solution). 15 µL proteinase K (10 mg/mL) and 5 μL RNase A (10 mg/mL) were added and lysis was performed overnight at 55 °C in a thermocycler at 500 rpm. 800 μL of extraction solution 1 (phenol:chloroform:isoamyl alcohol, 25:24:1) was added. After vortexing for 10 s and centrifugation (17000 × *g*, 4 °C, 10 min) the aqueous phase was transferred to a new 2 mL centrifuge tube. This extraction step was repeated with 700 µL extraction solution 2 (chloroform:isoamyl alcohol, 24:1). The aqueous phase was again transferred to a new tube and kept on ice from this point on. 1.2 mL of ice-cold ethanol was added to precipitate DNA. The DNA was dissolved in 250 µL DNase-free water, 25 µL of 3 M NaOAc was added and the DNA precipitated again with 250 µL ice-cold isopropanol. The yielded DNA was rinsed with ice-cold 70 % ethanol, dried at room temperature, and solved in 50 µL of DNase-free water. Concentration and purity of the yielded solutions were measured spectrophotometrically by a NanoDrop ND1000 photometer (ThermoScientific, Wilmington, NC, USA). Digestion into nucleosides was performed with 30 µg DNA per sample, spiked with 50 nmol of each ^15^*N*-labeled adduct and with 1 nmol of ^15^*N*_5_-dG according to the method described previously [48]. ^15^*N*-labeled DNA adducts were synthesized, purified, and characterized as described previously [14].

### LC-MS/MS analysis and quantification of DNA adducts

Mass-spectrometric measurement of DNA adducts was performed on an Agilent 1290 infinity UHPLC system consisting of a binary pump (G4220A), an autosampler (G4226A) and a column oven (G1316C) coupled with a Sciex QTrap 5500 MS. A C18-column (U-VDSpher PUR C18-E 1.8 μm; 50 × 4.6 mm) with corresponding guard column (U-VDSpher PUR C-18-E, 1.8 μm 5 × 4 mm) was used for separation. The eluent consisted of 0.1% aqueous acetic acid (A) and UHPLC grade methanol (B). Injection volume was 5 µL and flow rate was 0.8 mL/min. The following gradient was used: min 0–1: 10% B, min 1–1.20: 10–50% B, min 1.20–3.50: 50–80% B, min 3.50–3.51: 80–95% B, min 3.51–5.00: 95% B, min 5.00–5.01: 95–10% B, 5.01–7.00: 10% B. The column temperature was kept at 25 °C. The MS was operated in ESI+, MRM mode. Data acquisition and processing were carried out with the Analyst Software version 1.7.1 and Multiquant 2.0 Software (Sciex). Instrument-specific parameters were: ion spray voltage 5.5 kV; ion source temperature 500 °C; curtain gas 25 psi; nebulizer gas 65 psi; heater gas 60 psi; collisionally activated dissociation gas medium.

For calculation of the adduct levels, the respective dG concentrations in the samples were used and determined as reported previously [47]. For compound-specific mass spectrometric parameters please see supporting Table S1. Representative UPLC-MS/MS chromatograms are shown in supporting Fig. S9.

### Transient transfection with siRNA

Knockdown of SULT1A1 and p53 were performed using siGENOME SMARTpool siRNA purchased at Dharmacon (Lafayette, USA). Non-sense, scrambled RNA (Dharmacon) served as control. Transfections were essentially conducted as described previously [49] and successful knockdown was confirmed by western blot analysis as detailed below.

### Alkaline Comet assay

The alkaline Comet assay was generally performed as described [50]. Briefly, HepG2 cells were seeded in 3.5 cm dishes and grown overnight. Cells were incubated with increasing OH-ME concentrations for 14 h or 24 h. DMSO served as solvent control. As positive control, cells were exposed to 5 µM etoposide. Cells were harvested and diluted with medium (1 × 10^6^ cells/mL). Afterwards, 10 µL from the cell suspension were mixed with 120 µL low melting point agarose, transferred onto a slide pre-coated with agarose, and cooled for 5 min at 4 °C. The slides were then incubated for 1 h at 4 °C in lysis buffer (2.5 M sodium chloride, 10 mM TRIS pH 10, and 100 mM EDTA). Subsequently, the slides were transferred into the electrophoresis chamber and incubated with electrophoresis buffer (300 mM NaOH and 1 mM EDTA) for 20 min at 4 °C protected from light. After this unwinding step, electrophoresis was conducted for 20 min at 300 mA. The slides were then immersed in neutralization buffer (0.4 M TRIS pH 7.5), rinsed in H_2_O, fixed for 5 min in 100% ethanol, and air-dried for 2 h. The slides were finally stained with 50 µL propidium iodide solution and mounted with a coverslip. Slides were analyzed by fluorescence microscopy using Leica 6000 microscope equipped with the analysis software Leica Application Suite X. Comets were assessed with Image J and the plug-in Open Comet v1.3 (www.cometbio.org). At least 100 cells were analyzed per experiment.

### Isolation of RNA and quantitative real-time PCR

Preparation of total RNA, transcription into cDNA and quantitative gene expression analysis by Real Time PCR was performed as described [51]. The used primers are listed in supporting Table S2. In four independent experiments, qPCR was conducted in technical duplicates with a CFX96TM Real-Time PCR Detection System (Bio-Rad, München, Germany). The subsequent analysis was performed using CFX Manager^TM^ software. Non-transcribed controls were included in each run. Gene expression levels in human HepG2 and HCT116 cell lines were normalized to *ACTB* as well as *GAPDH* and the solvent control was set to one. In V79 and V79 CS cells, gene expression levels were normalized to hamster *ACTB* and *HSP90*.

### Immunofluorescence and confocal microscopy

Cells grown on cover slips were treated with OH-ME as indicated. DMSO served as solvent control, while N-OH-PhIP was included as positive control. Briefly, cells were fixed in ice-cold methanol at −20 °C for γH2AX staining or 4% paraformaldehyde (PFA) at room temperature for cytochrome c staining followed by blocking in PBS containing 5% (w/v) bovine serum albumin (BSA) and 0.3% (v/v) Triton X-100 for 1 h. Sample preparation for immunofluorescence staining of activated Bax included a fixation with 4% PFA followed by permeabilization with 0.2% CHAPS in PBS and a blocking step with PBS containing 5% (w/v) BSA. Immunofluorescence staining was essentially conducted as described [52] using the primary and secondary Alexa Fluor 488-conjugated antibodies listed in supporting Table S3. DNA was counterstained with TO-PRO-3 or DAPI (Life Technologies, Darmstadt, Germany) for 15 min. Finally, cells were mounted using VectaShield® (Vector Labs, Burlingame, CA) and analyzed by confocal microscopy using a Zeiss Axio Observer 7 microscope equipped with a 63x oil objective (Plan-Apochromat 63x/1.40 DIC M27) and a LSM900 confocal laser scanner (Zeiss, Oberkochen, Germany). Images were acquired by ZEN software version 3.4 (Zeiss) and processed using ImageJ software (NIH, MD).

### DNA fiber assay

The DNA fiber assay was performed as previously described [21]. HCT116 cells were challenged with OH-ME or N-OH-PhIP for 14 h, and HepG2 cells were treated for 24 h. Then, the cells were pulse-labeled with 25 µM 5-chloro-2’-deoxyuridine (CldU; Sigma, Steinheim, Germany) followed by labeling with 250 µM 5-Iodo-2’-deoxyuridine (IdU; TCI Deutschland, Eschborn, Germany). HCT116 cells were labeled for 30 min each pulse, while HepG2 cells were labeled for 40 min. Cells were then harvested and DNA fiber spreads were prepared as previously described [53, 54]. Fiber spreads were fixed in methanol:acetic acid mixture (3:1, v/v) and air-dried. Slides were rehydrated in H_2_O followed by incubation with 2.5 M HCl for 75 min for DNA denaturation. After neutralization, fiber spreads were incubated for 60 min in blocking buffer (5% goat serum + 1% bovine serum albumin (BSA) in PBS with 0.1% Tween-20. CldU was detected with a monoclonal rat anti-BrdU antibody (ab6326; Abcam, Berlin, Germany) and IdU by a monoclonal mouse anti-BrdU (#347580; Becton–Dickinson, UK) for 2.5 h, followed by donkey Cy3-coupled anti-rat and anti-mouse Alexa488-coupled fragment [ab´]2 specific secondary antibodies (both from Jackson ImmunoResearch Europe). Fibers were analyzed using a LSM 710 confocal laser-scanning microscope with ZEN 2009 software (Zeiss). CldU (red) and IdU (green) tracks were measured using LSM Image Browser (Zeiss) and micrometer values were converted into kilo base pairs by multiplying with a factor of 2.59. The following classes of labeled tracks were assessed and presented as percentage of all counted DNA tracks: green-red-green (1^st^ pulse origin), red-green (ongoing replication), red (stalled forks), red-green-red (termination), and green (2^nd^ pulse origin). A total of 150–300 forks from three independent experiments were analyzed.

### SDS-PAGE and western blot analysis

Cells were seeded in 3.5 cm or 6 cm dishes and allowed to adhere overnight. Cells were then exposed to OH-ME as indicated and harvested directly in 1× Lämmli loading buffer. Proteins were then separated by SDS-PAGE and transferred onto nitrocellulose membranes (Perkin Elmer, Rodgau, Germany) using a wet-blot chamber essentially as reported [55]. After blocking in 5% (w/v) non-fat dry milk in PBS/0.1% Tween-20, incubation with the primary antibody occurred overnight at 4 °C. Upon several washing steps, incubation with the corresponding secondary horseradish peroxidase-coupled antibody followed (Santa Cruz, Heidelberg, Germany) for 1 h. Using enhanced chemiluminescence and Western Lightning Plus-ECL (Perkin Elmer), proteins were detected using a c300 chemiluminescence imager (Azure biosystems, Dublin, USA) or a ChemiDoc MP imaging system (BioRad, München, Germany). The used primary and secondary antibodies are detailed in supporting table S4.

### Assessment of subG1 population by flow cytometry

SubG1 population indicative of apoptosis was assessed as described previously [23]. Cells were incubated with OH-ME as indicated. Cells were then collected, washed with PBS, and incubated in 80% ethanol at −20 °C overnight. Cells were washed with PBS, incubated with RNase A (Sigma) for 1 h, and stained by propidium iodide (Sigma). Finally, cells were analyzed by flow cytometry using a BD Accuri™ C6 (BD Biosciences, Heidelberg, Germany) and the corresponding software (BD Biosciences).

### Determination of cell viability by ATP, MTS, and AlamarBlue assay

Viability of cells was determined using the Cell Titer 96® AQueous One Solution Cell Proliferation Assay (Promega, Mannheim, Germany) as reported [56]. Cells were grown overnight in 96-well plates and then treated with increasing concentrations of OH-ME as indicated. DMSO served as solvent control. After 72 h, viability was measured using a microplate reader (Sunrise Tecan Reader, Crailsheim, Germany) according to the manufacturer’s instructions. Viability was also assessed with the CellTiter-Glo®Luminescent Cell Viability Assay (Promega, Mannheim, Germany), which measures the cellular ATP level. To this end, the cells were seeded in white 96-well-plates, treated as indicated, and incubated for 72 h. The assay was performed according to the manufacturer’s instructions in the multiwell reader Fluoroskan Ascent FL (Thermo Scientific). In addition, an AlamarBlue assay was performed. Cells were seeded in 96-well plates, treated as indicated, and incubated for 72 h. Incubation medium was exchanged with DMEM without supplements, containing 44 µM AlamarBlue and cells were incubated for 90 min. Cell viability was measured fluorometrically with excitation wavelength *λ* = 544 nm and emission wavelength λ = 590 nm (Fluoroskan Ascent FL, Thermo Scientific).

### Cell death measurement

Cell death induction was measured using Annexin V/PI staining and flow cytometry as described earlier [57]. Following treatment, attached and detached cells were harvested and washed with PBS. Cells were then stained with AnnexinV conjugated to Alexa Fluor 488 (Miltenyi Biotec, Bergisch Gladbach, Germany) for 15 min on ice in binding buffer (5% dye; 10 mM HEPES pH 7.4, 140 mM NaCl, 2.5 mM CaCl_2_, 0.1% BSA). Thereafter, propidium iodide (2% dye in binding buffer) was added and cells were analyzed on a BD Canto II flow cytometer (BD Biosciences). BD FACS Diva software 6.0 (BD Biosciences) was used for the gating of living cells (Annexin V/PI double negative), early apoptotic cells (Annexin V-positive, PI-negative), and late apoptotic/necrotic cells (AnnexinV/PI-double positive).

### Caspase 3/7 activity assay

Activation of caspase-3 and caspase-7 was studied using the Caspase-Glo® 3/7 Assay (Promega, Mannheim, Germany). To this end, cells seeded in white 96-well plates were treated with increasing OH-ME concentrations as indicated. After 24 h, cells were lysed and a luminogenic caspase-3/7 substrate was added according to the manufacturer’s instructions. Following an incubation period of 30 min, luminescence was assessed in a Fluoroskan Ascent FL 96-well plate reader (Thermo Scientific).

### Ethics

All animal experiments were approved by the government of Rhineland-Palatinate and the Animal Care and Use Committee at the TU Kaiserslautern. All animal studies were performed according to German federal law and the guidelines for the protection of animals.

### Statistics

Experiments were performed independently at least three times, except otherwise stated. Results from representative experiments are shown. Values underwent Grubbs’ test to exclude outliers and are displayed as mean + standard error of the mean (SEM) using the GraphPad Prism 8.0 Software (GraphPad Software Inc.). Statistical analysis was performed using two-sided Student’s *t*-test and statistical significance was defined as *p* < 0.05.

## Supplementary information

Supplementary figure legends

Supplementary tables

Supplementary figure S1

Supplementary figure S2

Supplementary figure S3

Supplementary figure S4

Supplementary figure S5

Supplementary figure S6

Supplementary figure S7

Supplementary figure S8

Supplementary figure S9

original western blot data

authorship contribution form

change of authorship request form

aj checklist

## Data Availability

The data generated during this study were included in the article and its supplementary files.
