# Supplementary material for: p53 triggers mitochondrial apoptosis following DNA damage-dependent replication stress by the hepatotoxin methyleugenol
Source: Cell Death Dis. 2022 Nov 29;13(11):1009. doi: 10.1038/s41419-022-05446-9 (PMC9708695; doi:10.1038/s41419-022-05446-9)
Supplement: Supplementary file 1 — Supplementary figure legends [file 41419_2022_5446_MOESM1_ESM.pdf]

Supplementary Figure Legends

Figure S1: Metabolic activation of methyleugenol and DNA adduct formation. ME is activated by CYP1A2 and additional CYP isoforms in the liver, which results in the formation of 1′-hydroxymethyleugenol (OH-ME) as main metabolite. OH-ME is conjugated with a sulfo-group in a reaction catalyzed predominantly by SULT1A1. The formed 1′-sulfoxymethyleugenol is instable and decomposes to a DNA-reactive carbocation under the release of sulphate. This yields N^2-MIE-dG as main DNA adduct, whereas N^6-MIE-dA represents the minor adduct.

Figure S2: Detection of SULT1A1 and measurement of DNA adducts by mass spectrometry. A SULT1A1 level in different cell lines used in this study. Cell lysates were analyzed by SDS-PAGE and western blot detection for SULT1A1. Hsp90 served as loading control. **B** and **C** Determination of N^2-MIE-dG and N^6-MIE-dA adducts in HCT116-p53$^{+/+}$ cells exposed to increasing concentrations of OH-ME for 24 h. Cells were harvested and genomic DNA was isolated followed by digestion to nucleosides. Adduct level was determined by UPLC-mass spectrometry using an internal isotope-labeled standard. Data are given as mean + SEM (n=3). Not significant, p> 0.05, **p<0.01, ***p<0.001. n.d. indicates “not detected”.

Figure S3: Analysis of γH2AX formation in hamster fibroblasts and HCT116 cells depending on SULT1A1 status. A Detection of γH2AX in V79 and V79 CS cells 24 h after exposure to 150 µM OH-ME or 50 µM PhIP. DMSO was used as solvent control. Representative confocal microscopy images are shown (scale bar: 10 µm). **B** The number of γH2AX foci per nucleus were determined by ImageJ software. Data are shown as mean + SEM (n=3, 50–100 cells per experiment). Ns: not significant, p>0.05, ****p<0.0001. **C** and **D** HCT116 cells proficient or deficient in p53 were treated for 24 h with increasing

concentrations of OH-ME as indicated. Formation of γH2AX foci were determined as described above. Representative confocal images are shown. Data are given as mean + SEM (n=3, typically 50 cells per experiment). Ns: not significant, p>0.05, **p>0.01, ***p>0.001. ****p<0.0001.

Figure S4: DNA damage and DNA damage response in liver cells and hamster fibroblasts. A Analysis of DNA strand break induction by 1′-OH-methyleugenol (OH-ME). HepG2 cells were exposed to increasing concentrations of OH-ME for 14 h and subject to the alkaline Comet assay. Etoposide was included as positive control. Representative images are shown. **B** Quantitative evaluation of alkaline Comet assay. Data are shown as mean + SEM (n=3). Ns: not significant, *p<0.05. **C** Formation of γH2AX foci in murine primary hepatocytes (mpH) after incubation with OH-ME for 24 h. Cells were fixed and processed for γH2AX immunostaining. Nuclei were counterstained with DAPI and images were acquired by confocal microscopy. Representative images are shown (size bar: 10 µm). **D** OH-ME induced DNA damage response in murine primary hepatocytes (mpH). Hepatocytes were incubated for 24 h with increasing OH-ME concentrations as indicated. p53 and γH2AX levels were monitored by SDS-PAGE and western blot analysis as described. Hsp90 was used as loading control. A representative western blot is depicted. **E** Time-dependent phosphorylation of CHK1 and H2AX in parental V79 cells and V79 CS cells after treatment with 150 µM OH-ME for up to 24 h. Samples were analyzed by SDS-PAGE and western blot detection of pCHK1 and γH2AX. Hsp90 served as loading control. **F** Phosphorylation of CHK1 serine 345 and H2AX in metabolically competent V79 CS cells after treatment with increasing concentrations of OH-ME (0–200 µM) for 24 h. 50 µM PhIP was included as positive control. Samples were analyzed by SDS-PAGE followed by western blot detection of pCHK1 and γH2AX. Hsp90 served as loading control.

Figure S5: Activation of the DNA damage response by ME-derived DNA adducts and replication stress in human HCT116 cells. **A** Concentration-dependent DNA damage

response in HCT116-p53$^{+/+}$ and HCT116-p53$^{-/-}$ cells triggered by OH-ME. Cells were challenged with up to 500 µM OH-ME for 24 h. Subsequently, cell lysates were analyzed by SDS-PAGE and western blot detection of pCHK1, γH2AX and p53. Hsp90 was detected as loading control. A representative western blot is shown. **B** - **D** Densitometric evaluation of p53, γH2AX and pCHK1 levels depending on the p53 status. Data are given as mean + SEM (n=4). *p<0.05, **p<0.01, ***p<0.001. **E** Impact of OH-ME on replication speed. HCT116 cells were treated for 14 h and replication speed was determined by DNA fiber assay using confocal microscopy. Data are given as mean + SEM (n=3). *p<0.05, **p<0.01. **F** Representative DNA fiber tracks of HCT116 cells exposed to OH-ME or N-OH-PhIP. **G** Distribution of replication structures assessed by the DNA fiber assay in HCT116 cells exposed to OH-ME or N-OH-PhIP for 14 h.

Figure S6: Knockdown of SULT1A1 and OH-ME triggered cytotoxicity in HepG2 cells. **A** Impact of SULT1A1 knockdown on OH-ME triggered cell death. HepG2 cells were transfected with SULT1A1 siRNA or scrambled siRNA (scrRNA) and then challenged with OH-ME as indicated. Cell death induction was measured after 72 h by Annexin V-FITC/PI staining and flow cytometry. Representative dot plots are shown. **B** Impact of SULT1A1 knockdown on cell morphology upon OH-ME treatment. Cells were transfected as described above and then treated with OH-ME as indicated. After 72 h, cell morphology was assessed by phase contrast microscopy.

Figure S7: Analysis of cell death induction in HepG2 liver cells. **A** Time-dependent expression of anti-apoptotic genes (*c-IAP1* and *c-IAP2*) in HepG2 cells treated with 75 µM OH-ME or solvent control for up to 48 h. Gene expression was assessed by qPCR (n=4). Data are depicted as mean + SEM. **B** Expression of the anti-apoptotic genes *Survivin* and *BCL-2* in HepG2 cells exposed to 75 µM OH-ME or solvent control for 24 h. Gene expression was assessed by qPCR (n=3). Data are depicted as mean + SEM. **C**

Assessment of Bax activation in HepG2 cells after treatment with 50 µM OH-ME for 48h. Cells were fixed, processed and immunostained for activated BAX, while nuclei were visualized by DAPI. Images were acquired by confocal microscopy followed by quantitative evaluation of Bax-positive cells (n=3). Data are shown as mean + SEM. ****p>0.0001. **D** Western blot analysis of caspase-8 cleavage in HepG2 cells exposed for 24 h to increasing concentrations of OH-ME (0–250 µM). Treatment with 10 µM etoposide (ETO) served as positive control. Hsp90 was detected as loading control.

Figure S8: Cytotoxicity of OH-ME in HepG2 and HCT116 cells depending on the p53 status. **A** HepG2 cells were transfected with p53 siRNA or scrambled RNA followed by treatment with increasing concentrations of OH-ME for 24 h. Expression of p53 and its downstream target p21 were then analyzed by SDS-PAGE and western blot detection. Hsp90 served as loading control. **B** Isogenic HCT116 cells proficient or deficient for p53 were challenged with increasing OH-ME concentrations for 72 h. 5-FU served as positive control. Cell death induction was assessed by AnnexinV-FITC/PI staining and flow cytometry. Representative dot blots are depicted. **C** HCT116 cells proficient or deficient for p53 were treated as described above. Cell morphology was assessed by phase contrast microscopy. **D** Pharmacological inhibition of p53 and impact on OH-ME triggered cytotoxicity. HepG2 cells were pre-incubated with the p53 inhibitor pifithrin-α (Pif-α) for 2 h or left untreated. Cells were then challenged with increasing concentrations of OH-ME for 48 h and viability was determined using the Resazurin assay. Data are shown as mean + SEM (n=4). ****p<0.0001.

Figure S9: DNA adduct analysis by mass spectrometry. UPLC-MS/MS chromatograms of a digested DNA sample from HepG2 cells exposed to 0 or 75 µM OH-ME for 24 h. The chromatograms of N^2-MIE-dG (m/z = 428 → 328) and N^6-MIE-dA (m/z = 428 → 177) are shown. The indicated transitions were used as quantifier.
