# Supplementary material for: p53 triggers mitochondrial apoptosis following DNA damage-dependent replication stress by the hepatotoxin methyleugenol
Source: Cell Death Dis. 2022 Nov 29;13(11):1009. doi: 10.1038/s41419-022-05446-9 (PMC9708695; doi:10.1038/s41419-022-05446-9)
Supplement: Supplementary file 2 — Supplementary tables [file 41419_2022_5446_MOESM2_ESM.pdf]

Supplementary Tables

Table S1: Compound specific mass spectrometric parameters[a] for dG, $^{15}N_5$-dG, N^2-MIE-dG, N^6-MIE-dA, [$^{15}N_5$]- N^2-MIE-dG and [$^{15}N_5$]- N^6-MIE-dA.

	Q1	Q3	DP	FP	EP	CEP	CE	CXP
dG	268.175	151.9[b]	6	350	4.5	12	15	18
	268.175	134.9	6	350	4.5	12	47	20
$^{15}N_5$-dG	273.083	157.3[b]	26	370	4	20	17	4
	273.083	138.9	26	370	4	20	49	4
N^2-MIE-dG	444.166	328.1 [b]	46		10		17	34
	444.166	177.1	46		10		33	12
N^6-MIE-dA	428.170	177.0 [b]	71		10		35	14
	428.170	312.1	71		10		21	22
[$^{15}N_5$]- N^2-MIE-dG	449.078	333.1 [b]	61		10		15	24
	449.078	177.1	61		10		41	12
[$^{15}N_5$]- N^6-MIE-dA	433.094	177.1 [b]	56		10		33	12
	433.094	317.1	56		10		21	18

[a] Q1, quadrupole 1 (m/z); Q3, quadrupole 3 (*m/z*); DP, declustering potential (V); FP, focusing potential; EP, entrance potential (V); CEP, collision cell entrance potential (V); CE, collision energy (V); CXP, cell exit potential (V).

[b] Transitions used as quantifier.

Table S2: Primers used for qPCR

qPCR target	Forward Primer (5′-3′)	Reverse primer (5′- 3′)
ACTB	TGGCATCCACGAAACTACC	GTGTTGGCGTACAGGTCTT
ACTB hamster	CAAGGCCAACCGTGAAAA	ACCAGAGGCATACAGGGACA
BAX	CAGAAGGCACTAATCAAG	ATCAGATGTGGTCTATAATG
BCL2	TTCAGAGACAGCCAGGAGAAA	AGTACCTGAACCGGCACCT
c-IAP1	TTCCCAGGTCCCTCGTATCA	CCGGCGGGGAAAGTTGAATA
c-IAP2	TCACTCCCAGACTCTTTCCA	CCCCGTGTTCTACAAGTGTC
FASR	AGAACTTGGAAGGCCTGCAT	CTGGTTCATCCCCATTGACT
GAPDH	CATGAGAAGTATGACAACAG	ATGAGTCCTTCCACGAT
HSP90 hamster	GCAGTACGCCTGGGAGTC	CCGTTTGGTCTTCTTTCAGG
MDM2	ATCTTGATGCTGGTGTAA	AGGCTATAATCTTCTGAGTC
NOXA	ACACGGTGGACGTCCTGT	ACGAAGCACTTGGGGAAGAT
p21	TACATCTTCTGCCTTAGT	TCTTAGGAACCTCTCATT
PUMA	GACGACCTCAACGCACAGTA	CTGGGTAAGGGCAGGAGTC
SULT1A1	CAAAGGATGTGGCAGTTTCC	CCGACCATGAACTTCTCCAG
Survivin	ATGACTTGTGTGTGATGA	GTTTGTGCTATTCTGTGAA

Table S3: Antibodies used for confocal immunofluorescence microscopy

Antibody	Catalog No.	Provider
Anti-cytochrome C, monoclonal mouse	#12963	Cell Signaling Technology, Danvers, Massachusetts, USA
Anti-BAX (2D2), monoclonal mouse	#89477	Cell Signaling Technology, Danvers, Massachusetts, USA
Anti-BrdU antibody, rat monoclonal	ab6326	Abcam, Cambridge, UK
Anti-BrdU antibody, mouse monocloanl	#347580	Becton–Dickinson, UK
Anti-γH2AX, monoclonal rabbit	ab11174	Abcam, Cambridge, UK
F(ab')2-Goat-anti-Mouse IgG (H-L) Alexa Fluor 488	A-11017	Life Technologies, Darmstadt, Germany
Goat anti-Rabbit IgG (H+L) Alexa Fluor 488	A-11008	Life Technologies, Darmstadt, Germany
Donkey Cy3-coupled anti-rat	712-165-150	Jackson ImmunoResearch Europe
Donkey Alexa488-coupled anti-mouse	715-545-150	Jackson ImmunoResearch Europe

Table S4: Antibodies used for SDS-PAGE and Western blot analysis

Antibody	Catalog No.	Provider
Anti-p53 (1C12), monoclonal mouse	#2524	Cell Signaling Technology, Danvers, Massachusetts, USA
Anti-p53 (DO1), monoclonal mouse	sc-126	Santa Cruz Biotechnology, Heidelberg, Germany
Anti-p21 (C19), polyclonal rabbit	sc-397	Santa Cruz Biotechnology, Heidelberg, Germany
Anti-p21 (F-5), monoclonal mouse	sc-6246	Santa Cruz Biotechnology, Heidelberg, Germany
Anti-Hsp90α/β, monoclonal mouse	sc-13119	Santa Cruz Biotechnology, Heidelberg, Germany
Anti-pChk1 serine 345, monoclonal rabbit	#2348T	Cell Signaling Technology, Danvers, Massachusetts, USA
Anti-pChk2 threonine 68, monoclonal rabbit	#2197T	Cell Signaling Technology, Danvers, Massachusetts, USA
Anti-cleaved Caspase 3, monoclonal rabbit	#9661	Cell Signaling Technology, Danvers, Massachusetts, USA
Anti-Caspase 8 p18, rabbit polyclonal	Sc-7890	Santa Cruz Biotechnology, Heidelberg, Germany
Anti-cleaved Caspase 9, monoclonal rabbit	#7237	Cell Signaling Technology, Danvers, Massachusetts, USA
Anti-SULT1A1, polyclonal rabbit	GTX55811	Genetex, Irvine, California, USA
Anti-γH2AX, monoclonal rabbit	ab11174	Abcam, Cambridge, UK
Goat-anti-Mouse HRP (IgGκ binding protein)	sc-516102	Santa Cruz Biotechnology, Heidelberg, Germany
Goat-anti-Rabbit-HRP	#7074S	Cell Signaling Technology, Danvers, Massachusetts, USA
