# Supplementary figures and images for: p53 triggers mitochondrial apoptosis following DNA damage-dependent replication stress by the hepatotoxin methyleugenol
Source: Cell Death Dis. 2022 Nov 29;13(11):1009. doi: 10.1038/s41419-022-05446-9 (PMC9708695; doi:10.1038/s41419-022-05446-9)

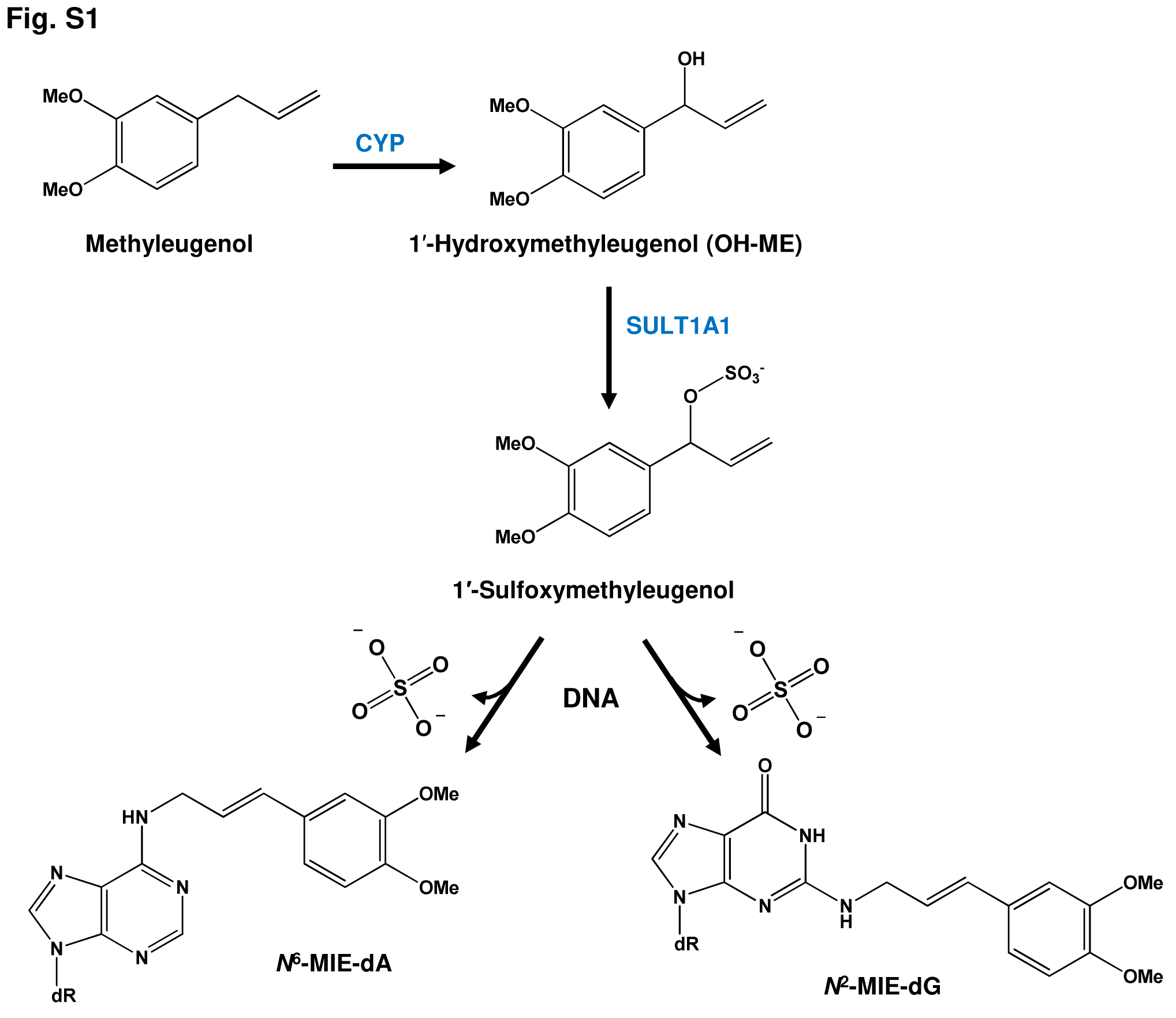

Supplement: Supplementary file 3 — Supplementary figure S1 [file 41419_2022_5446_MOESM3_ESM.tif]

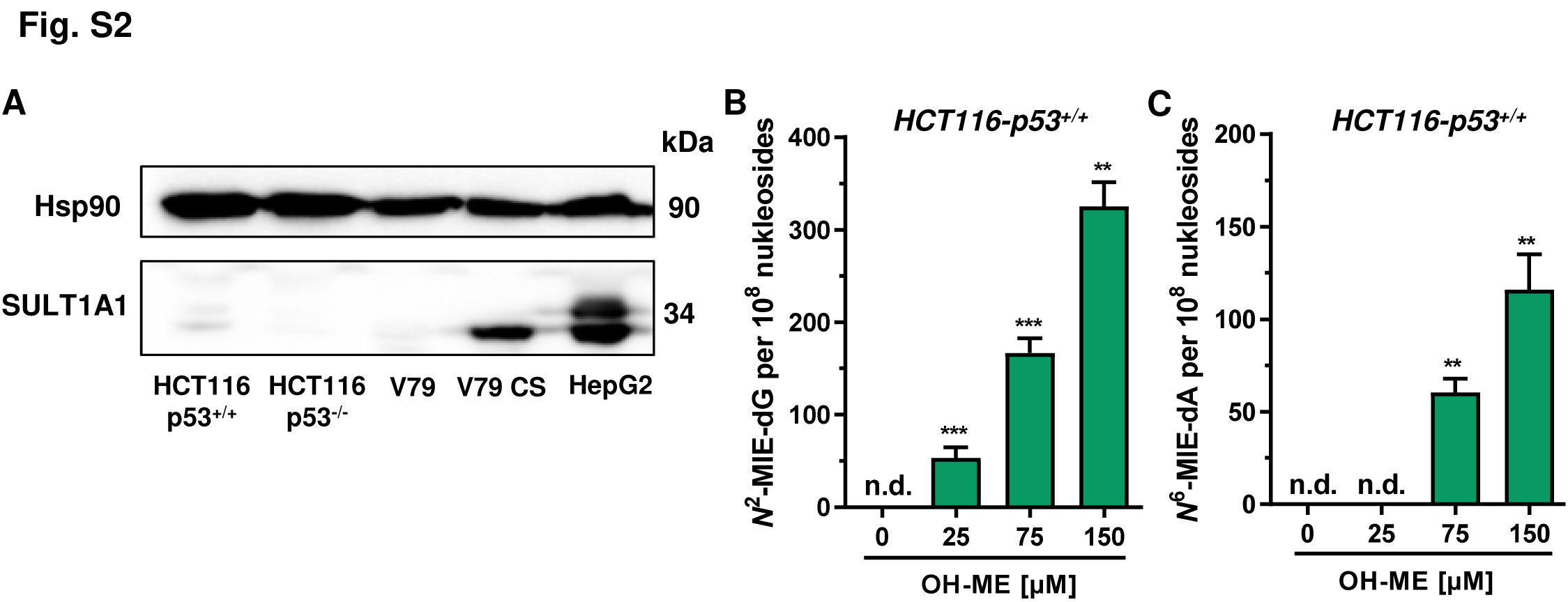

Supplement: Supplementary file 4 — Supplementary figure S2 [file 41419_2022_5446_MOESM4_ESM.tif]

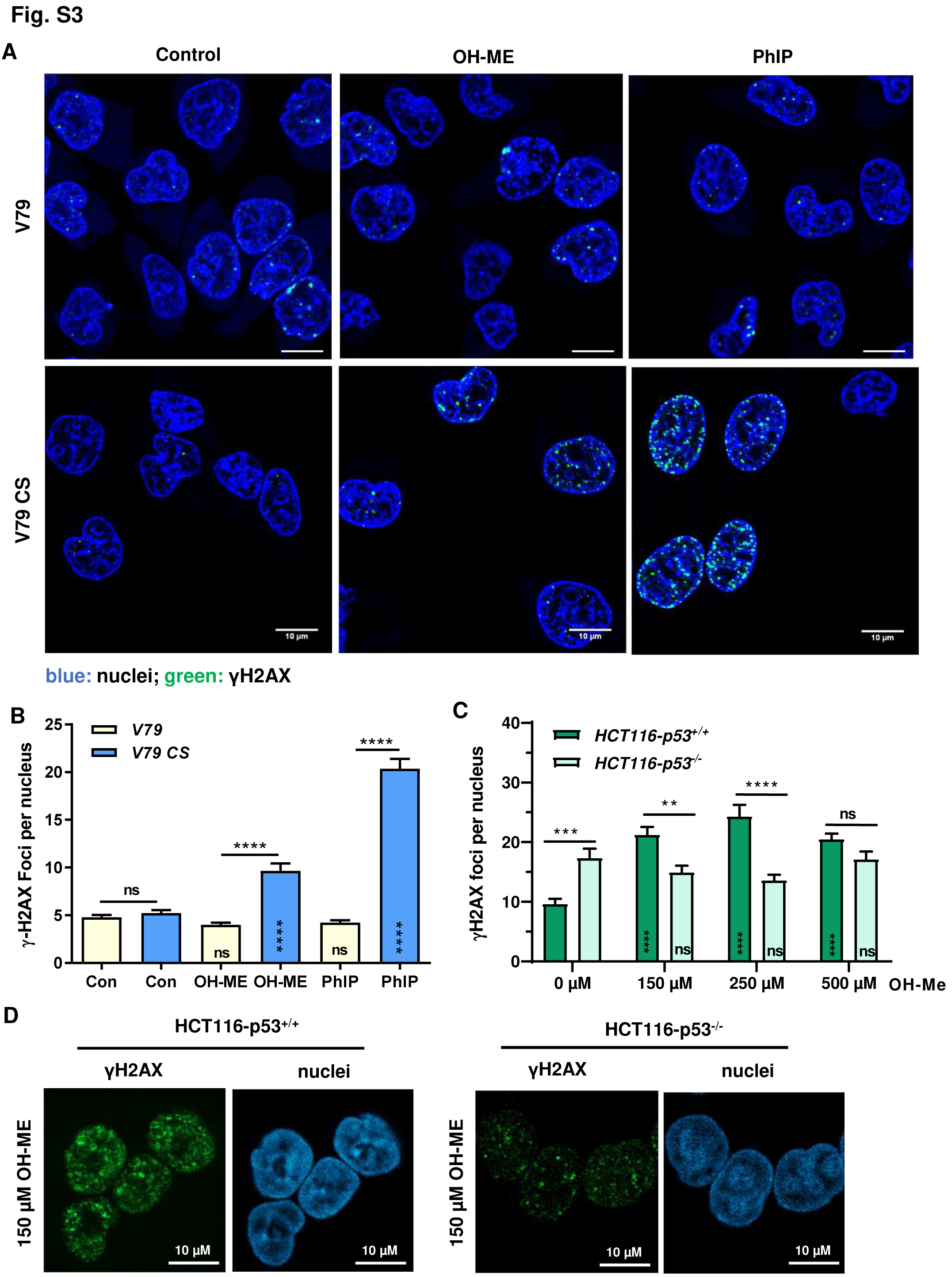

Supplement: Supplementary file 5 — Supplementary figure S3 [file 41419_2022_5446_MOESM5_ESM.tif]

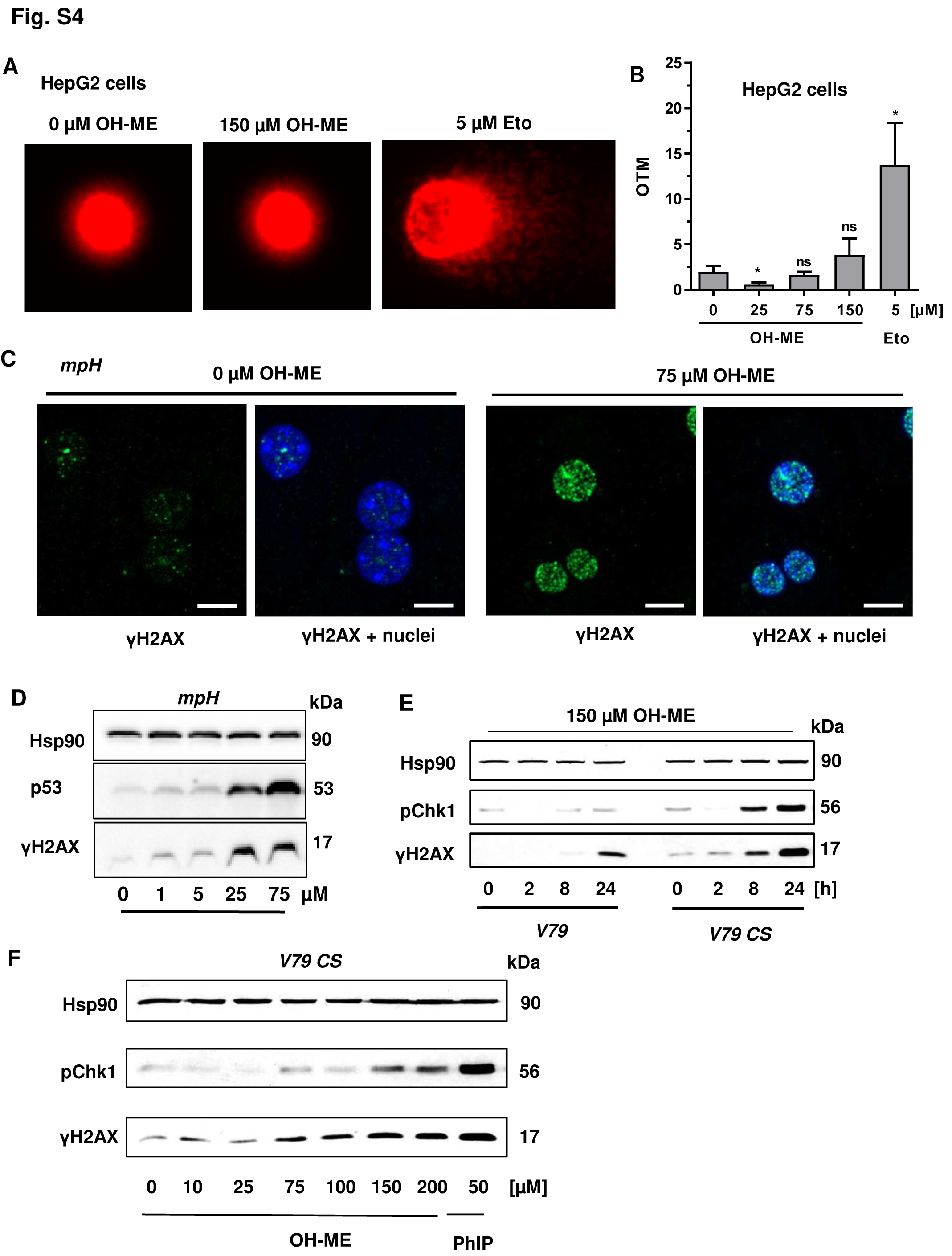

Supplement: Supplementary file 6 — Supplementary figure S4 [file 41419_2022_5446_MOESM6_ESM.tif]

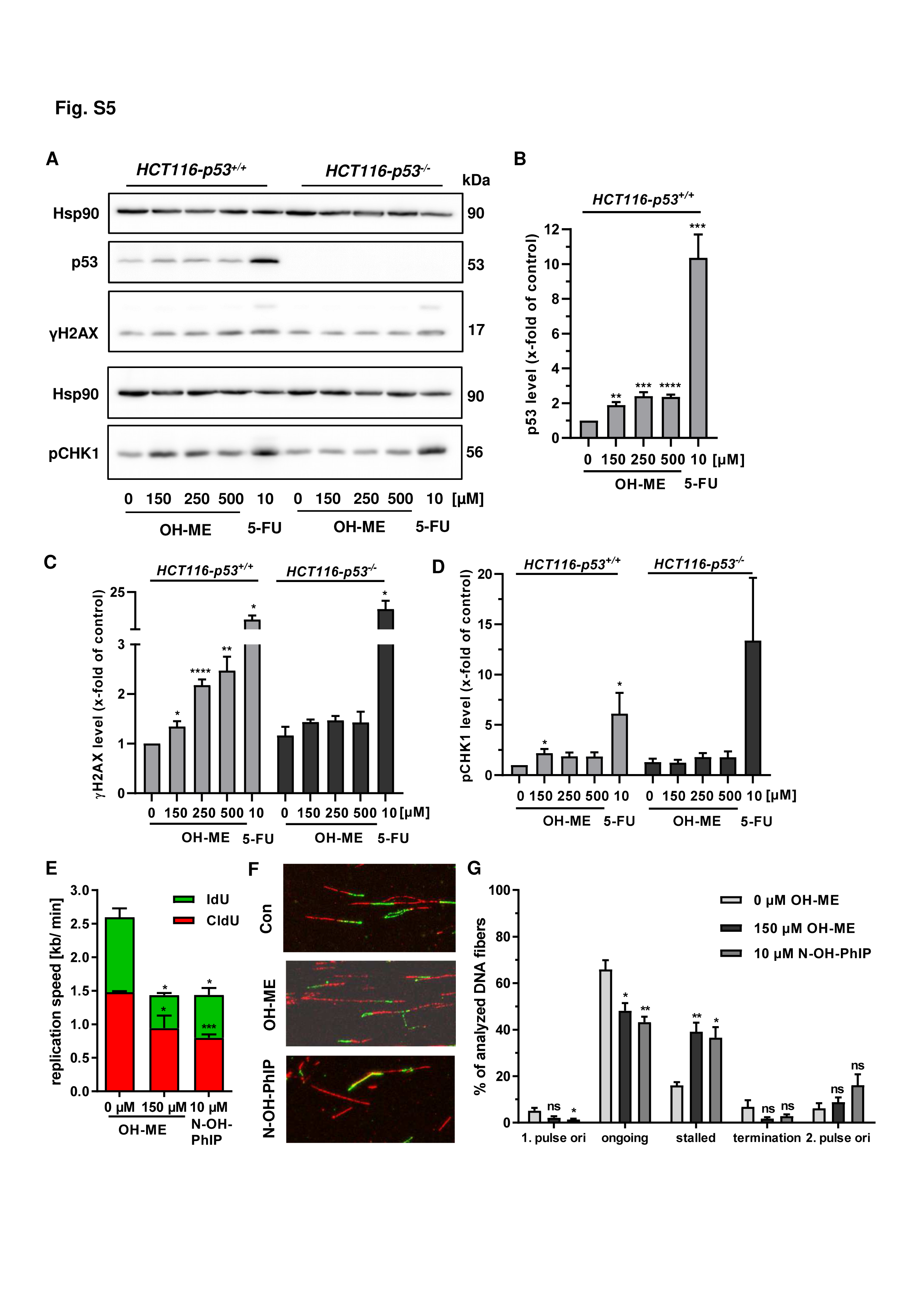

Supplement: Supplementary file 7 — Supplementary figure S5 [file 41419_2022_5446_MOESM7_ESM.tif]

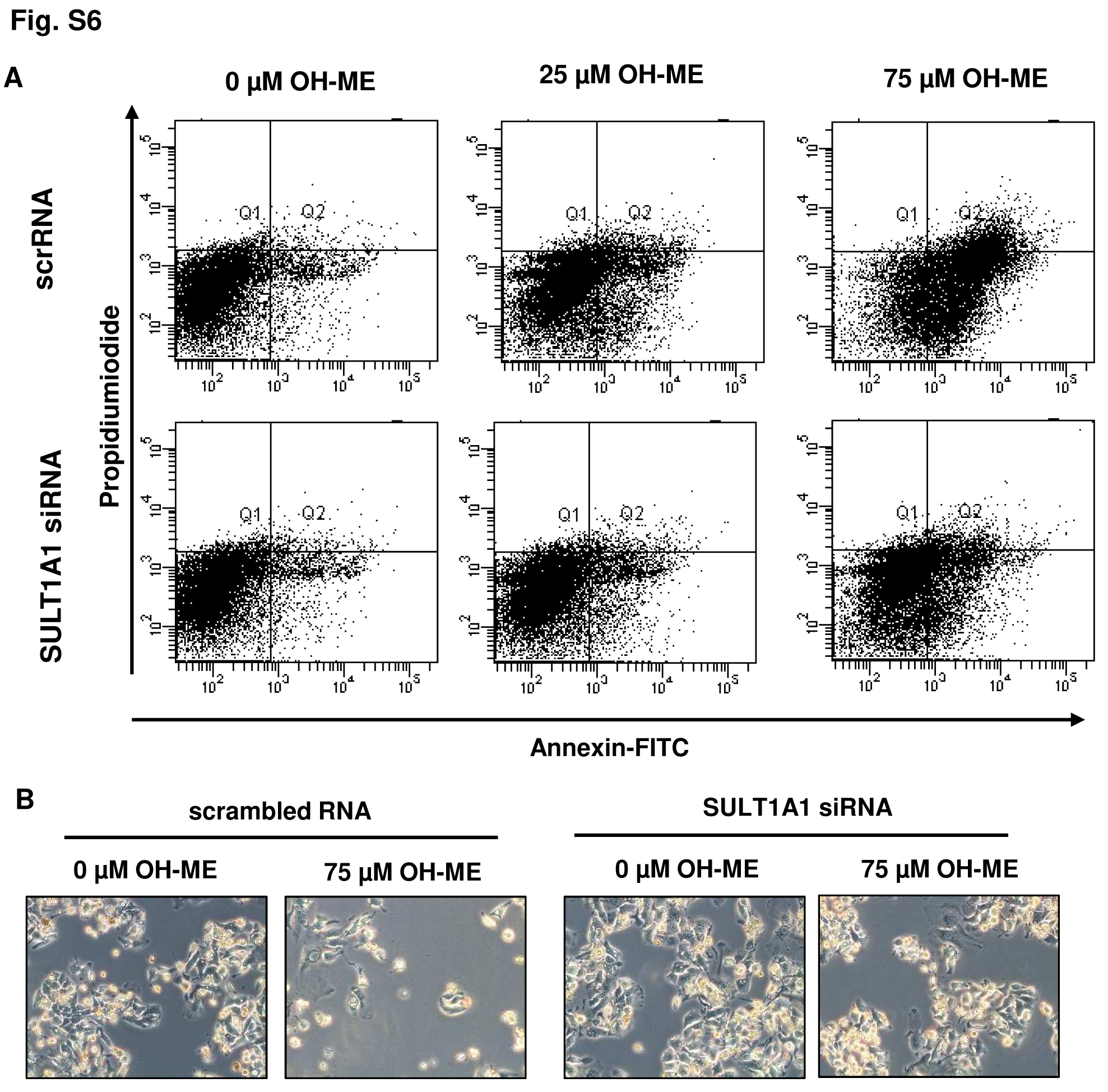

Supplement: Supplementary file 8 — Supplementary figure S6 [file 41419_2022_5446_MOESM8_ESM.tif]

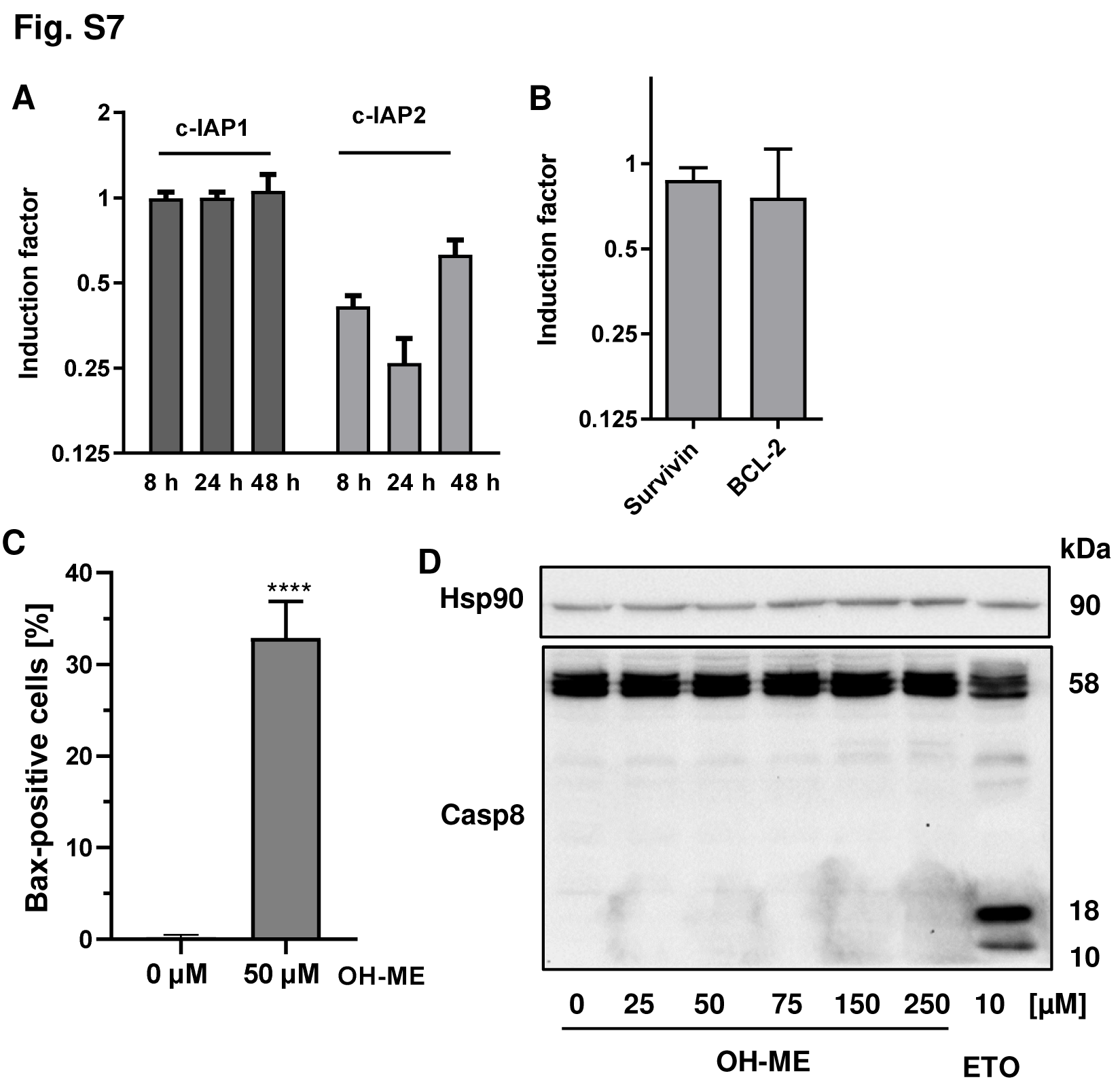

Supplement: Supplementary file 9 — Supplementary figure S7 [file 41419_2022_5446_MOESM9_ESM.tif]

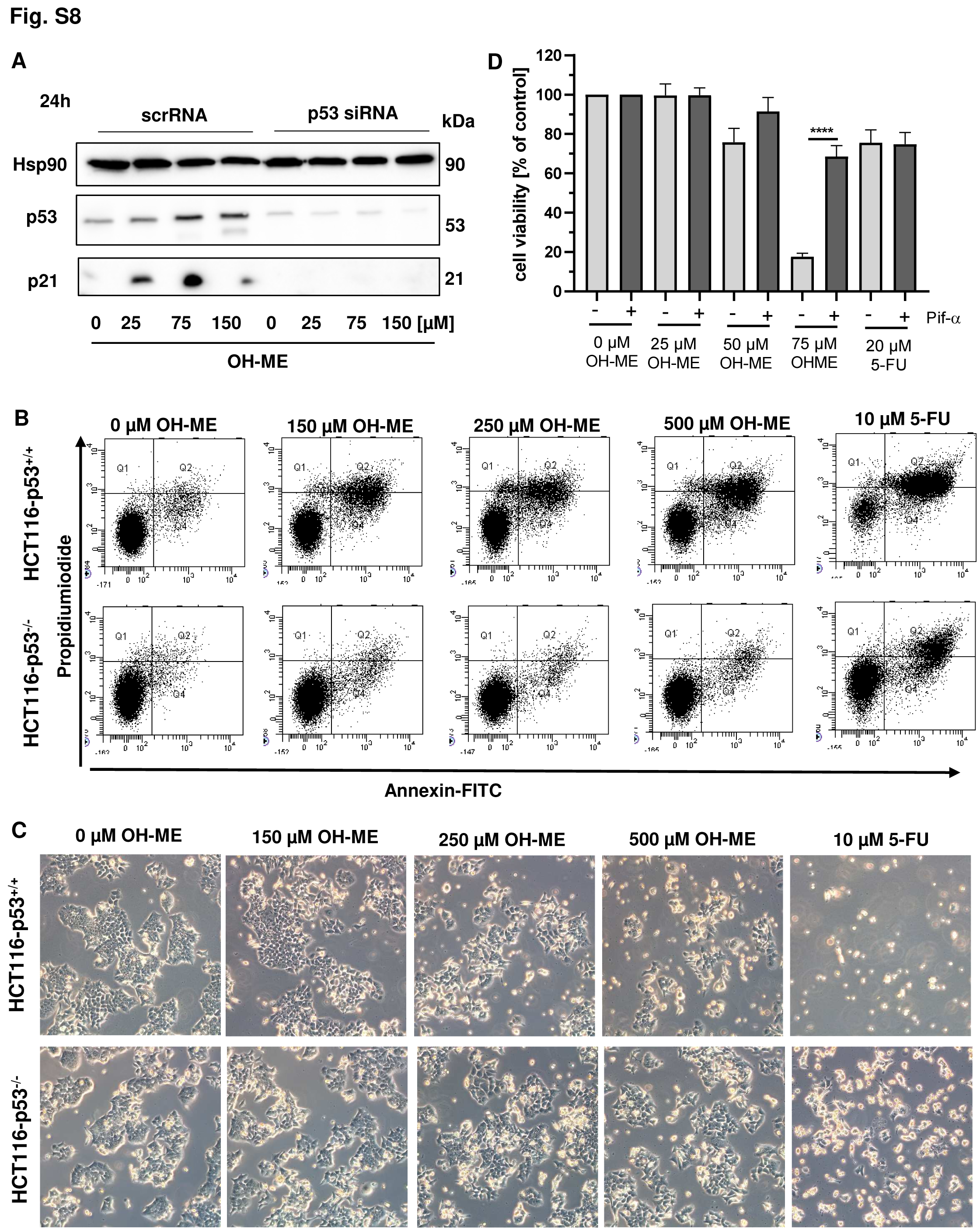

Supplement: Supplementary file 10 — Supplementary figure S8 [file 41419_2022_5446_MOESM10_ESM.tif]

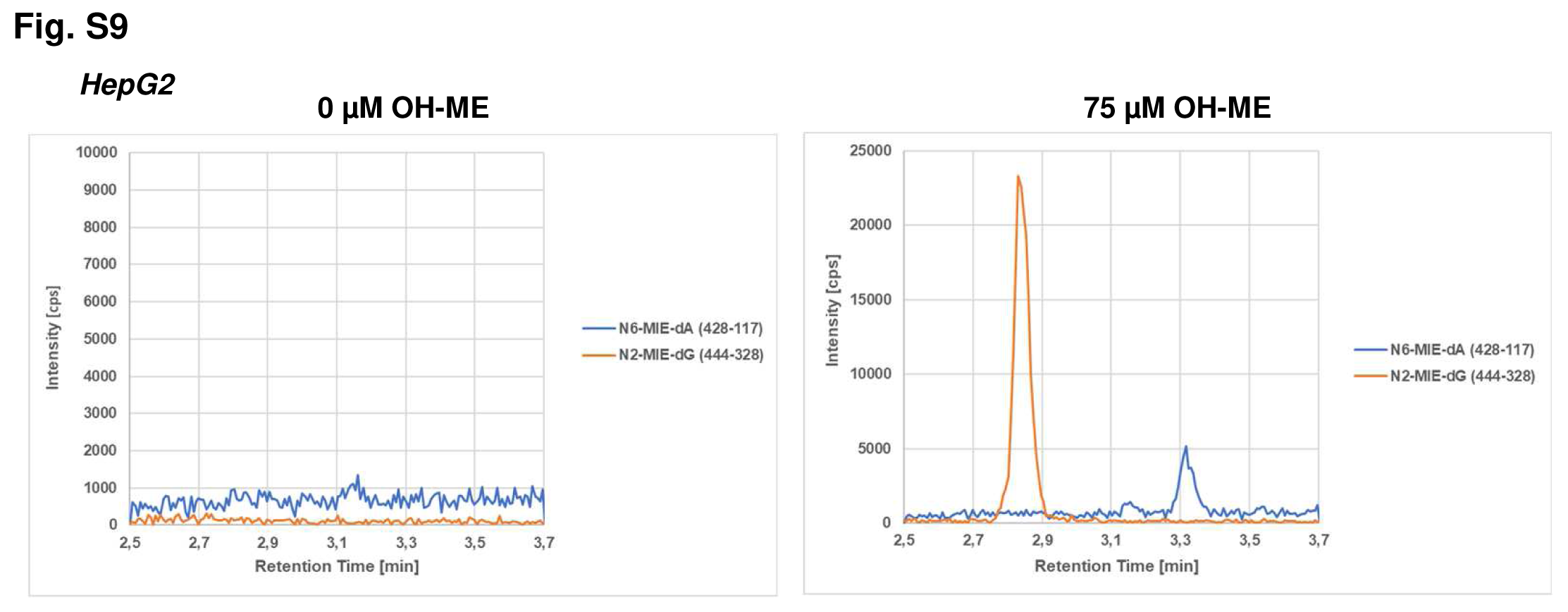

Supplement: Supplementary file 11 — Supplementary figure S9 [file 41419_2022_5446_MOESM11_ESM.tif]

**Fig. 2A**

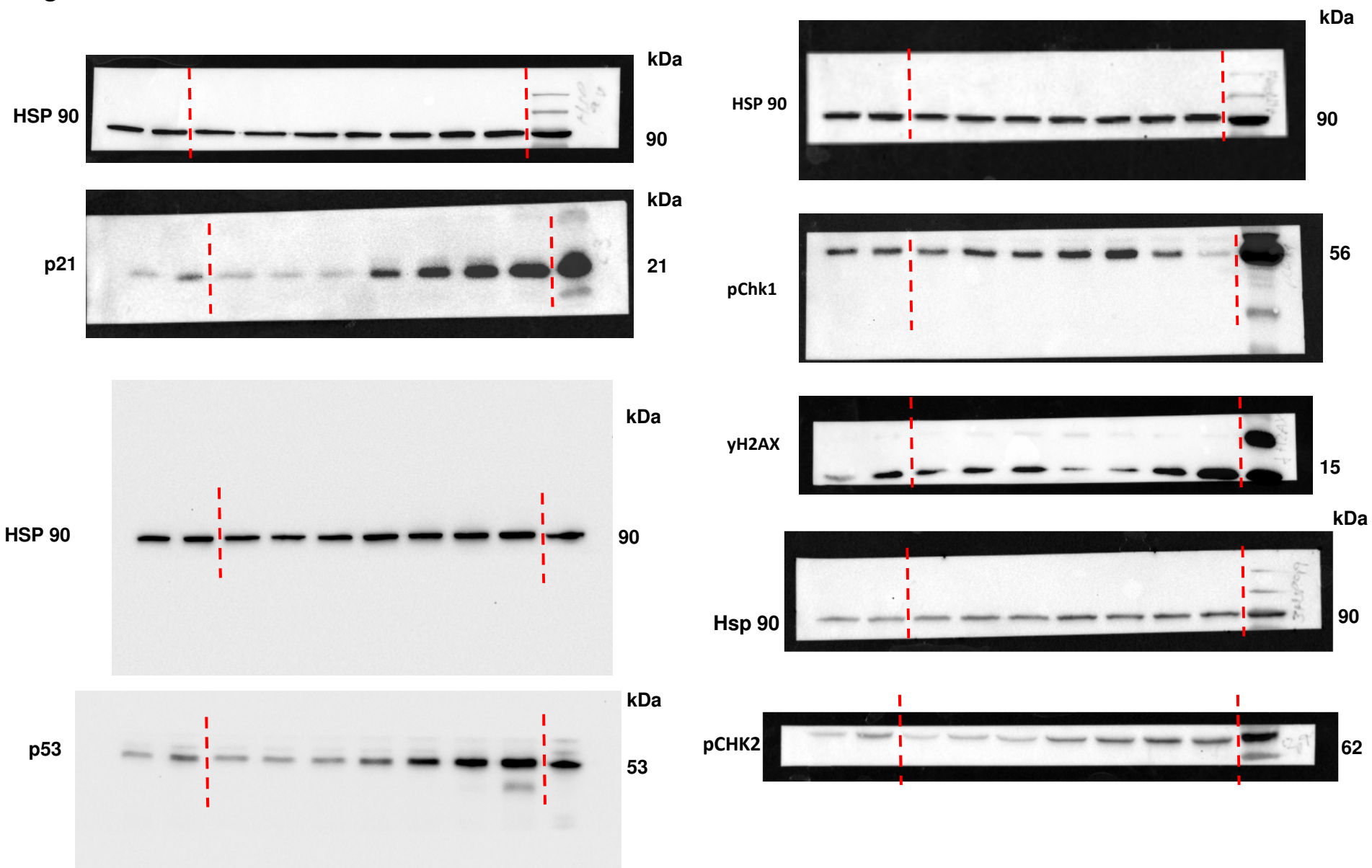

**Fig. 2B**

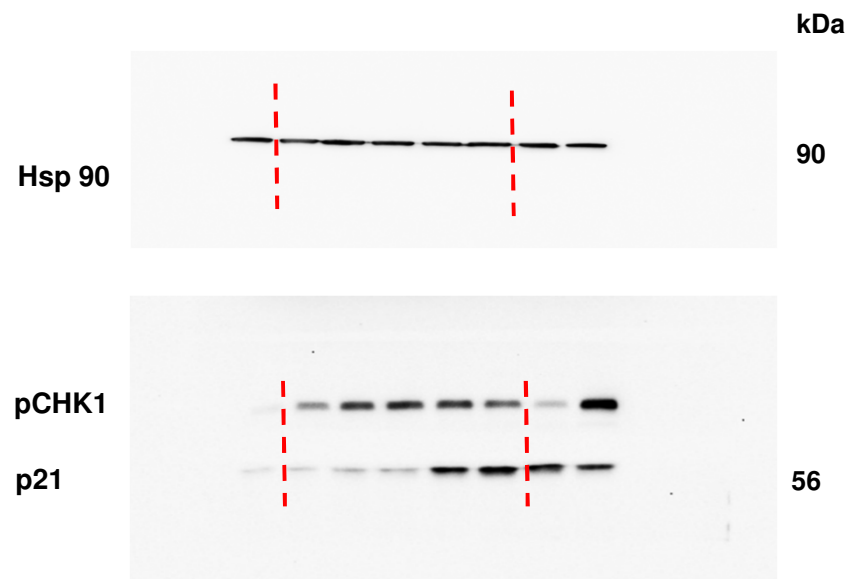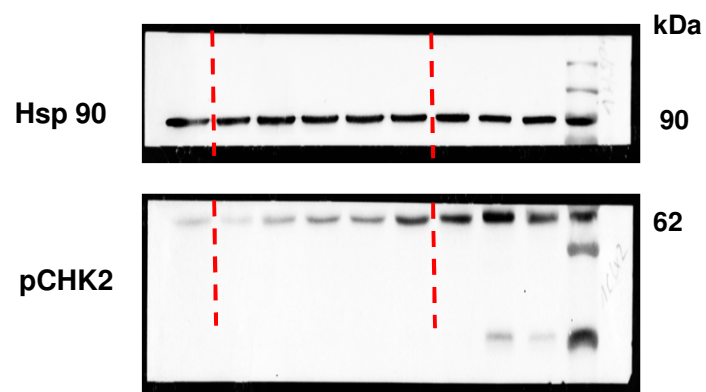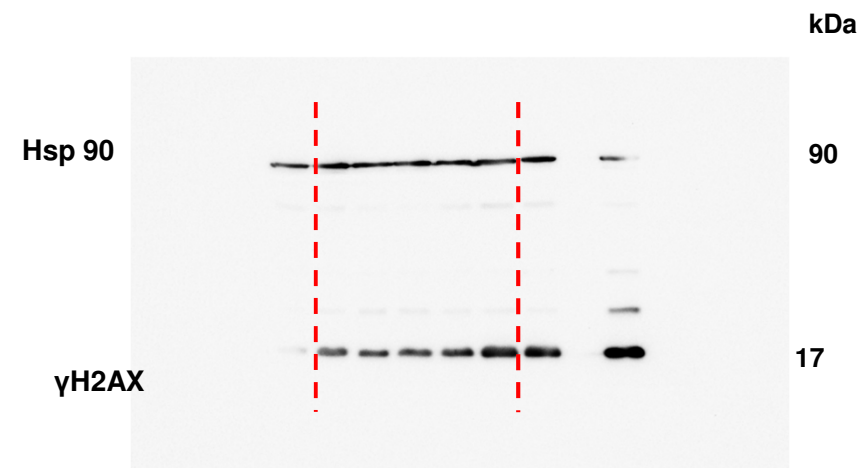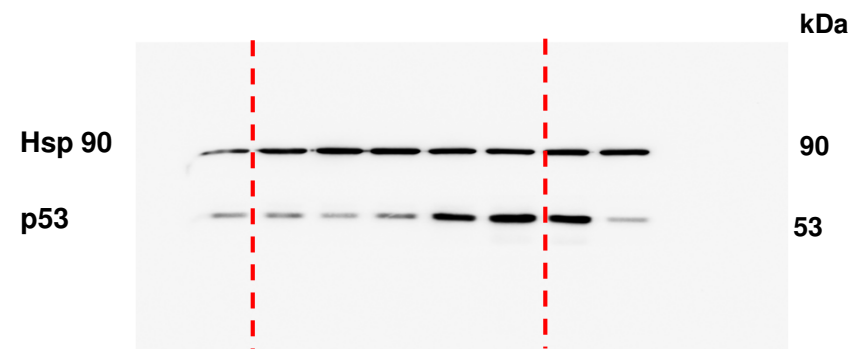

Fig. 3G

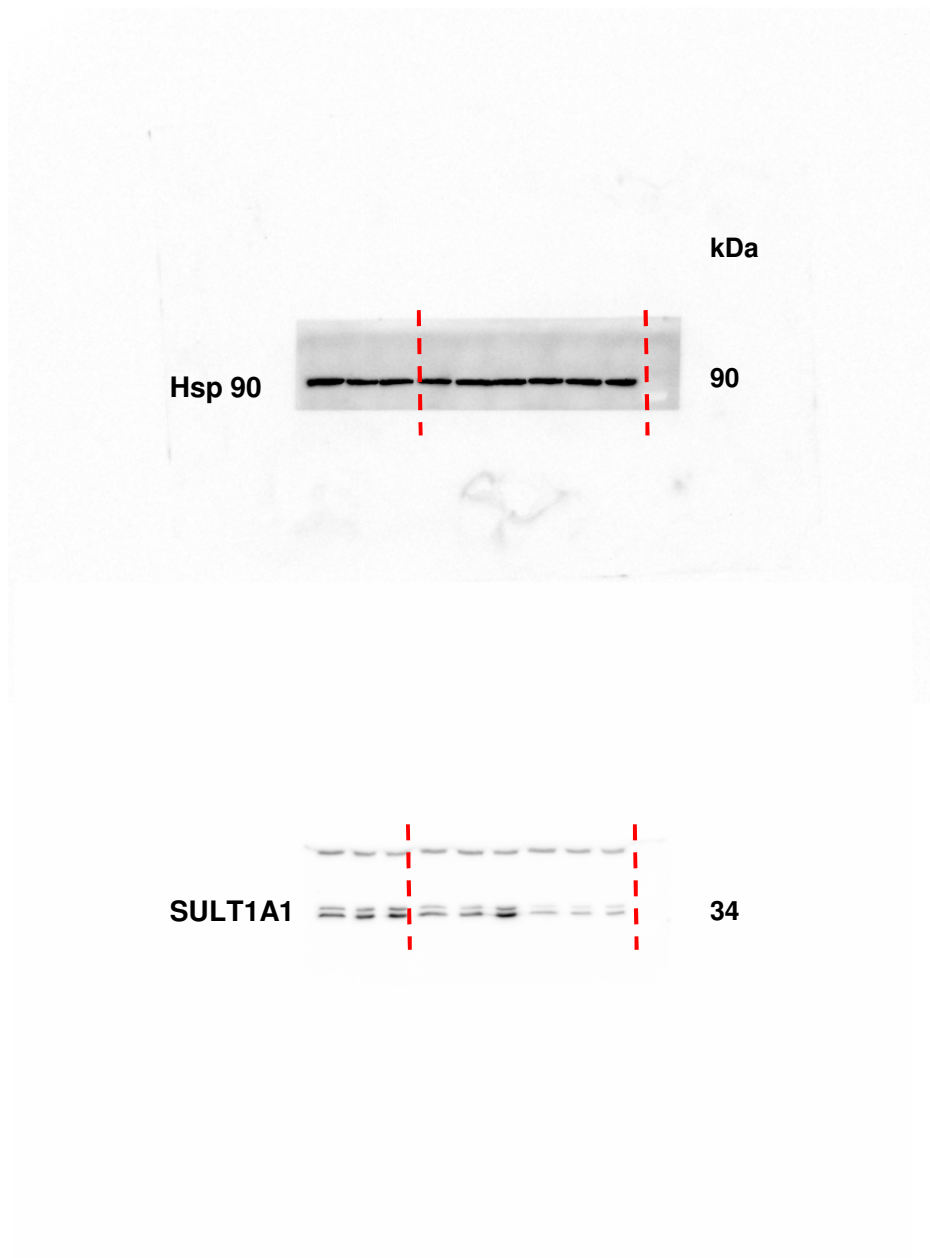

Fig. 4D

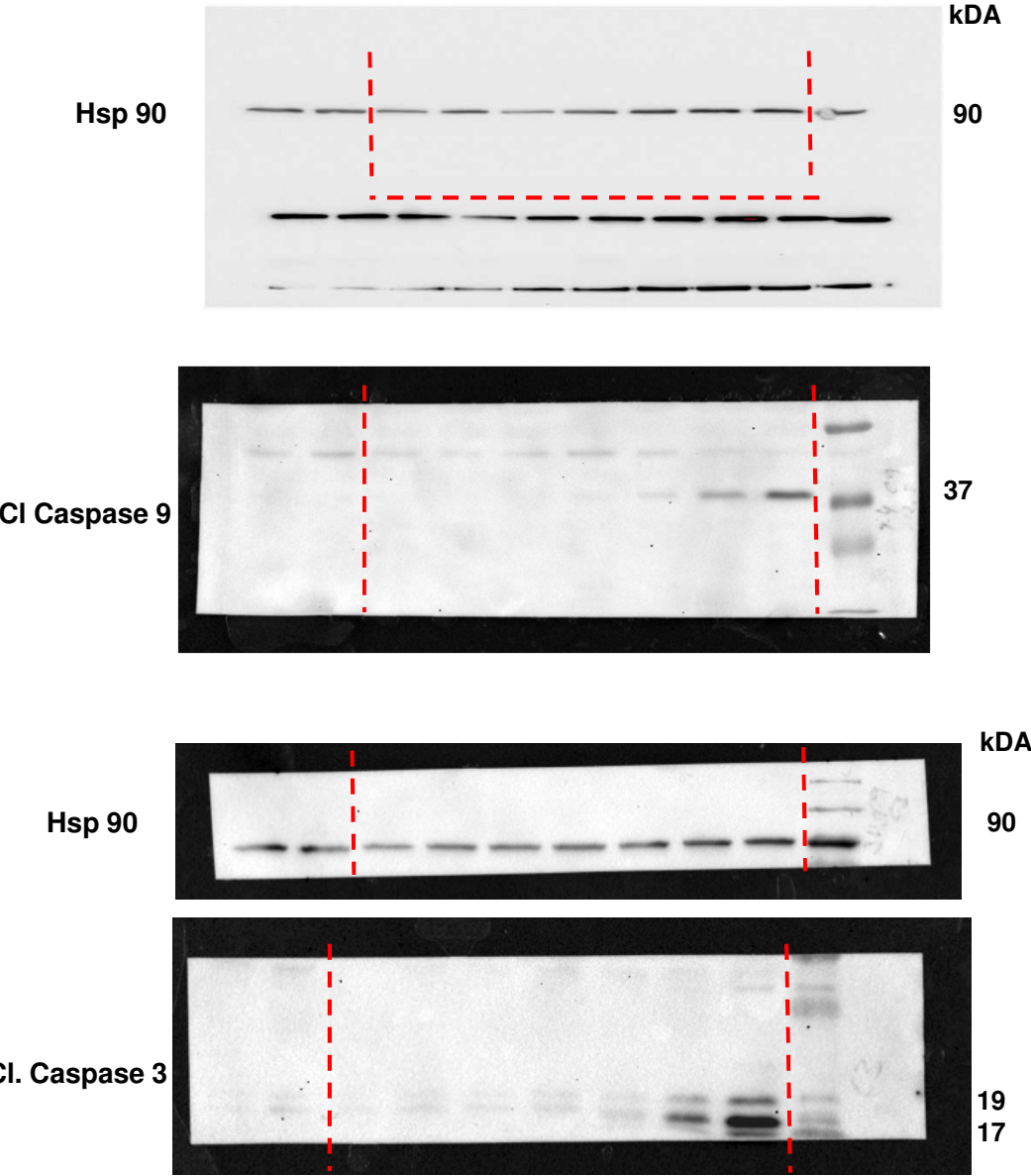

**Fig. 5A**

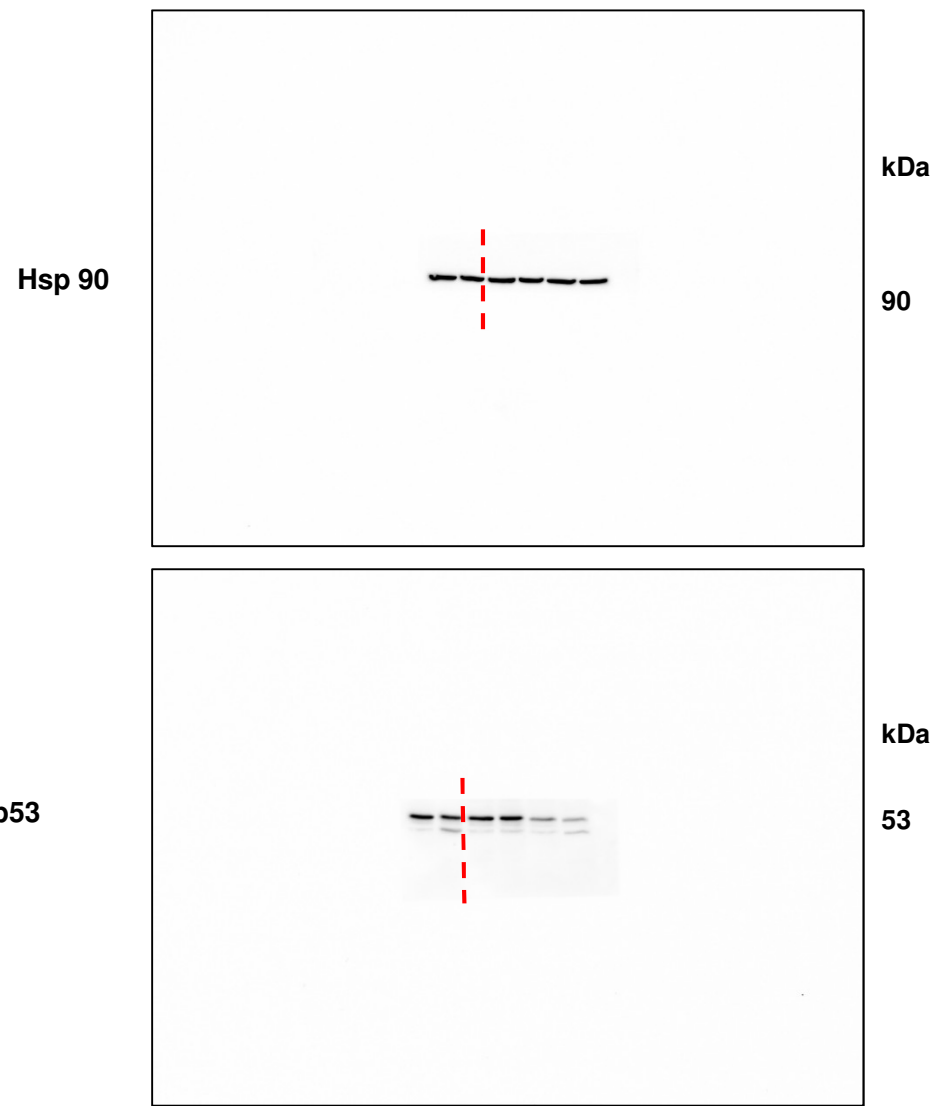

**Fig. 5F**

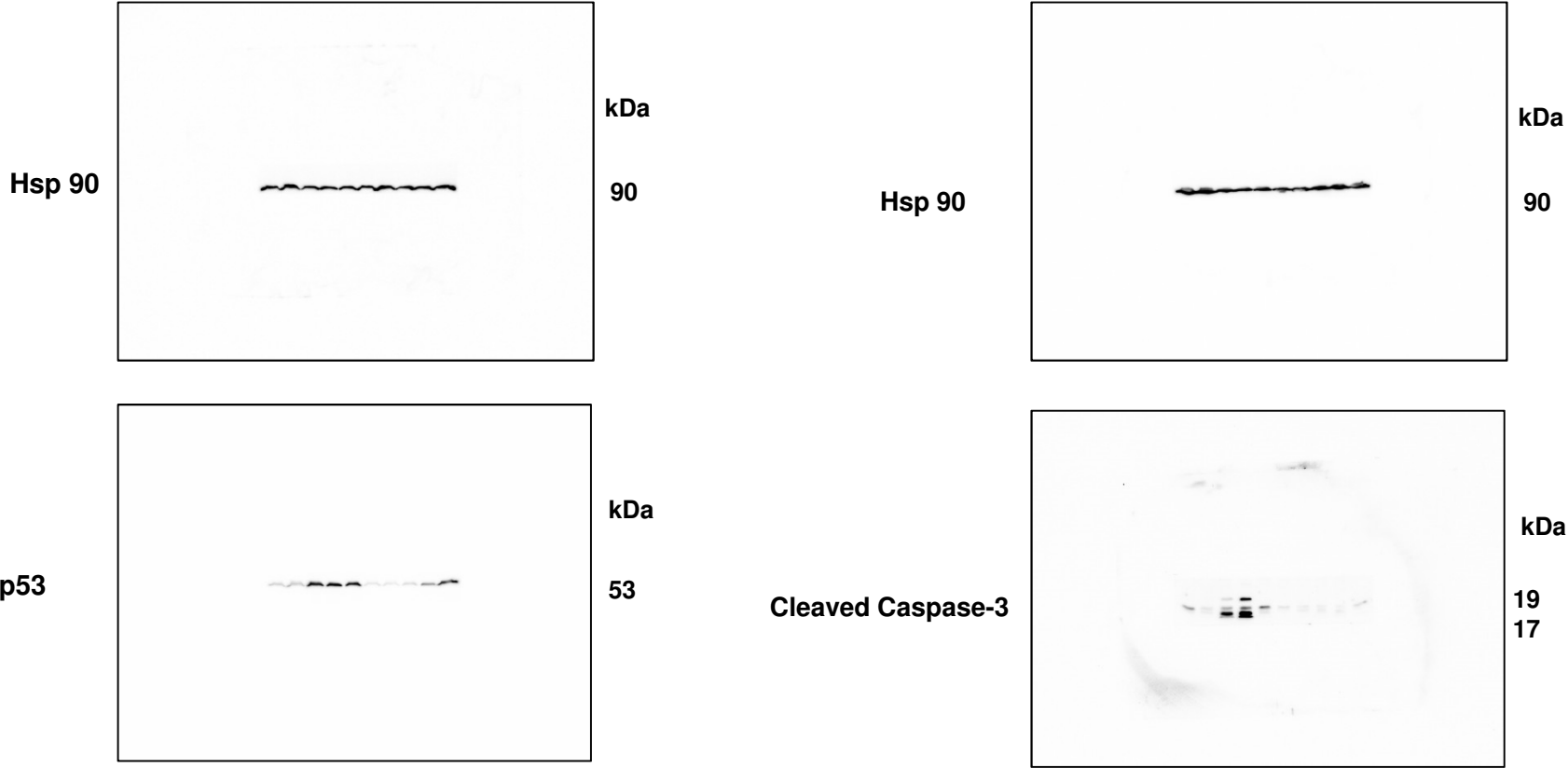

**Fig. S2B**

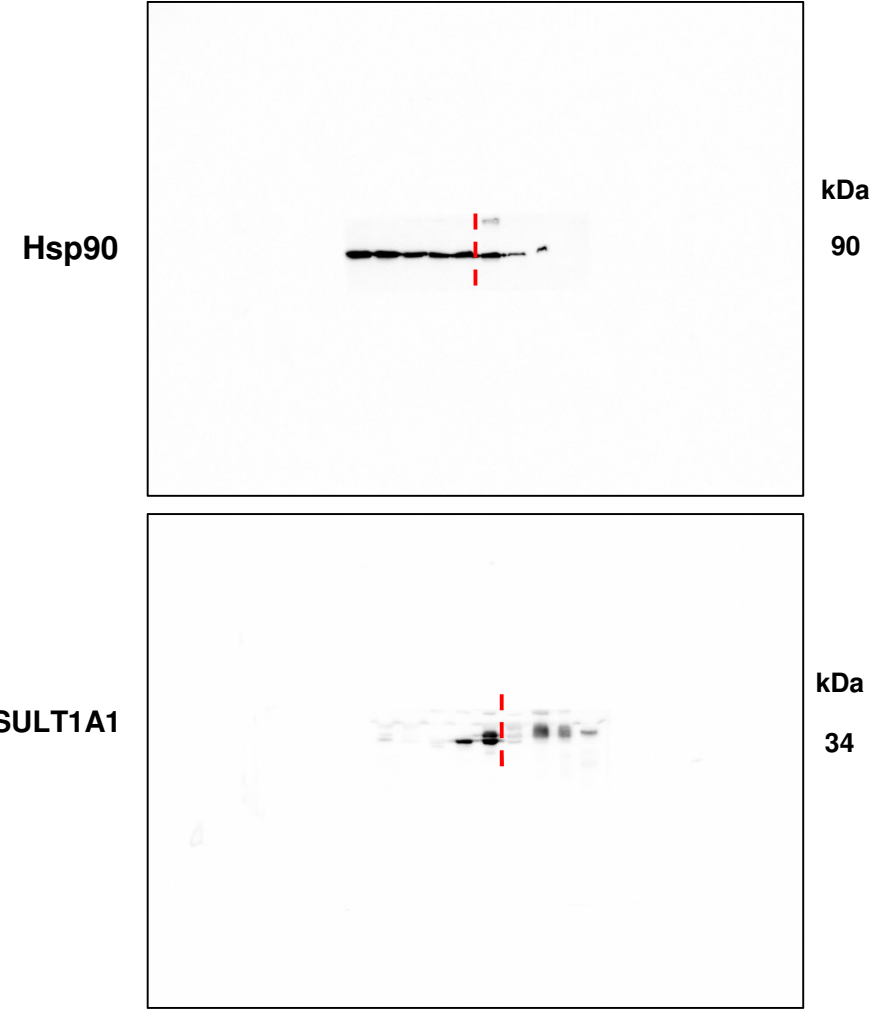

**Fig. S4D**

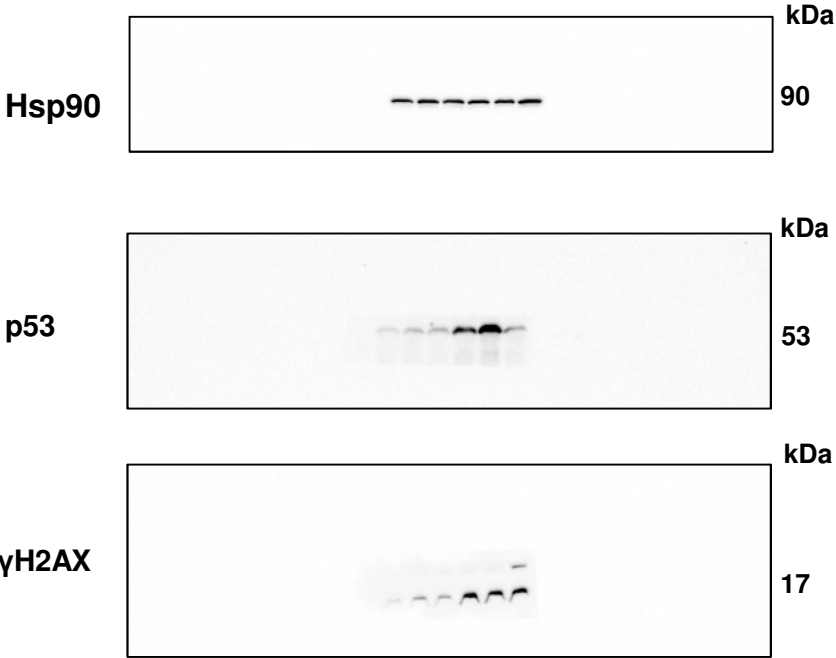

Fig. S4E

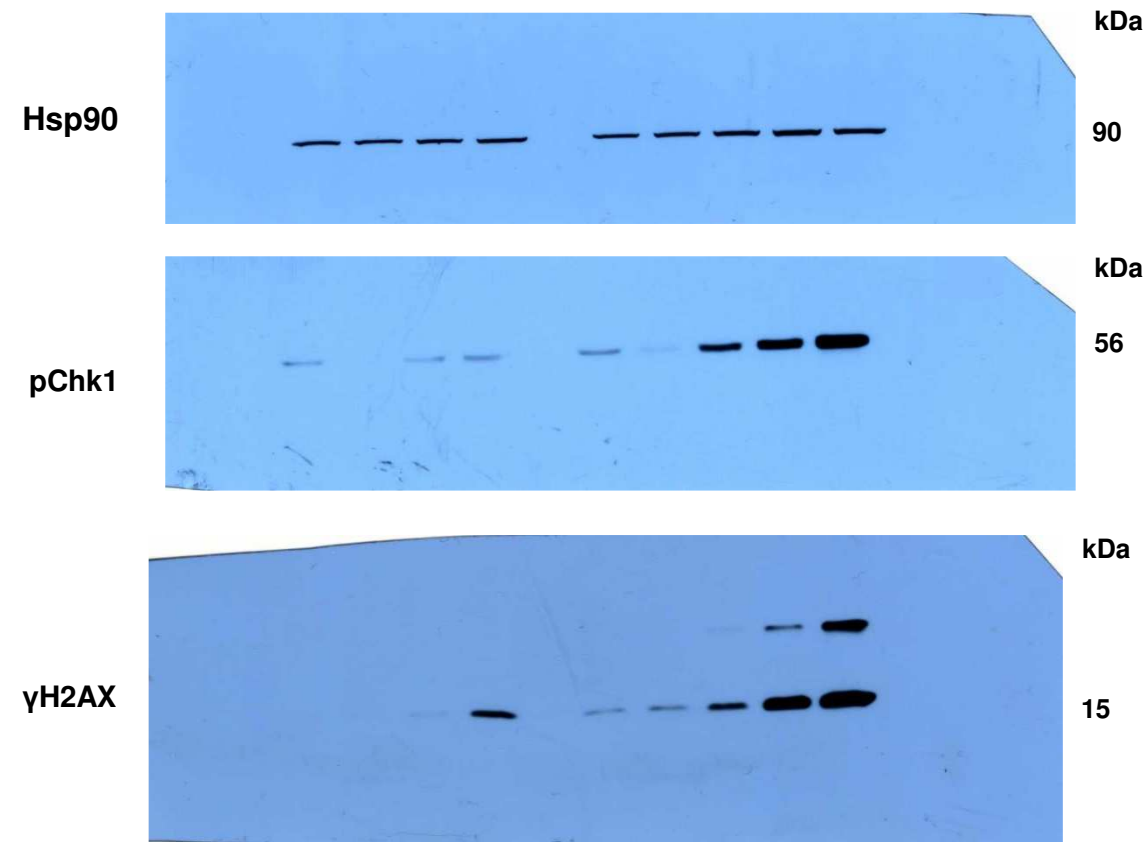

**Fig. S4F**

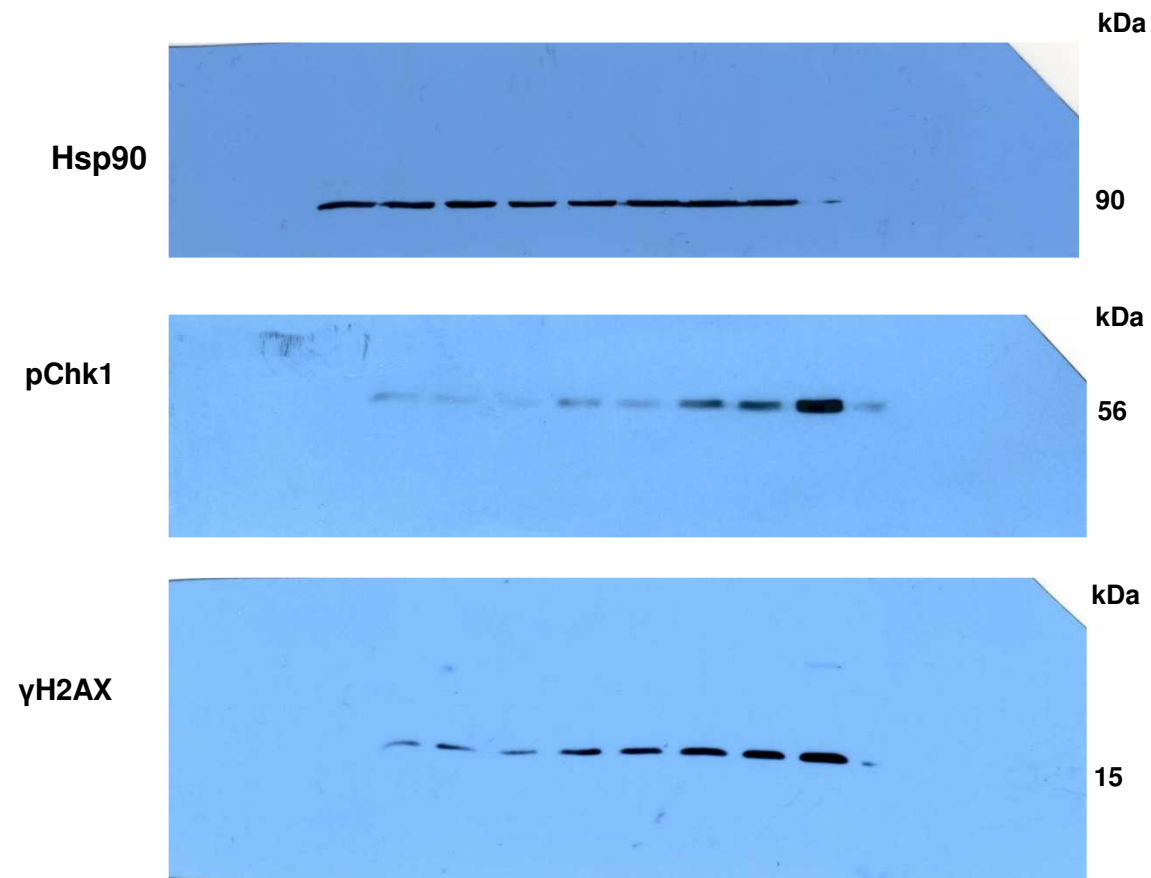

Fig. S5A

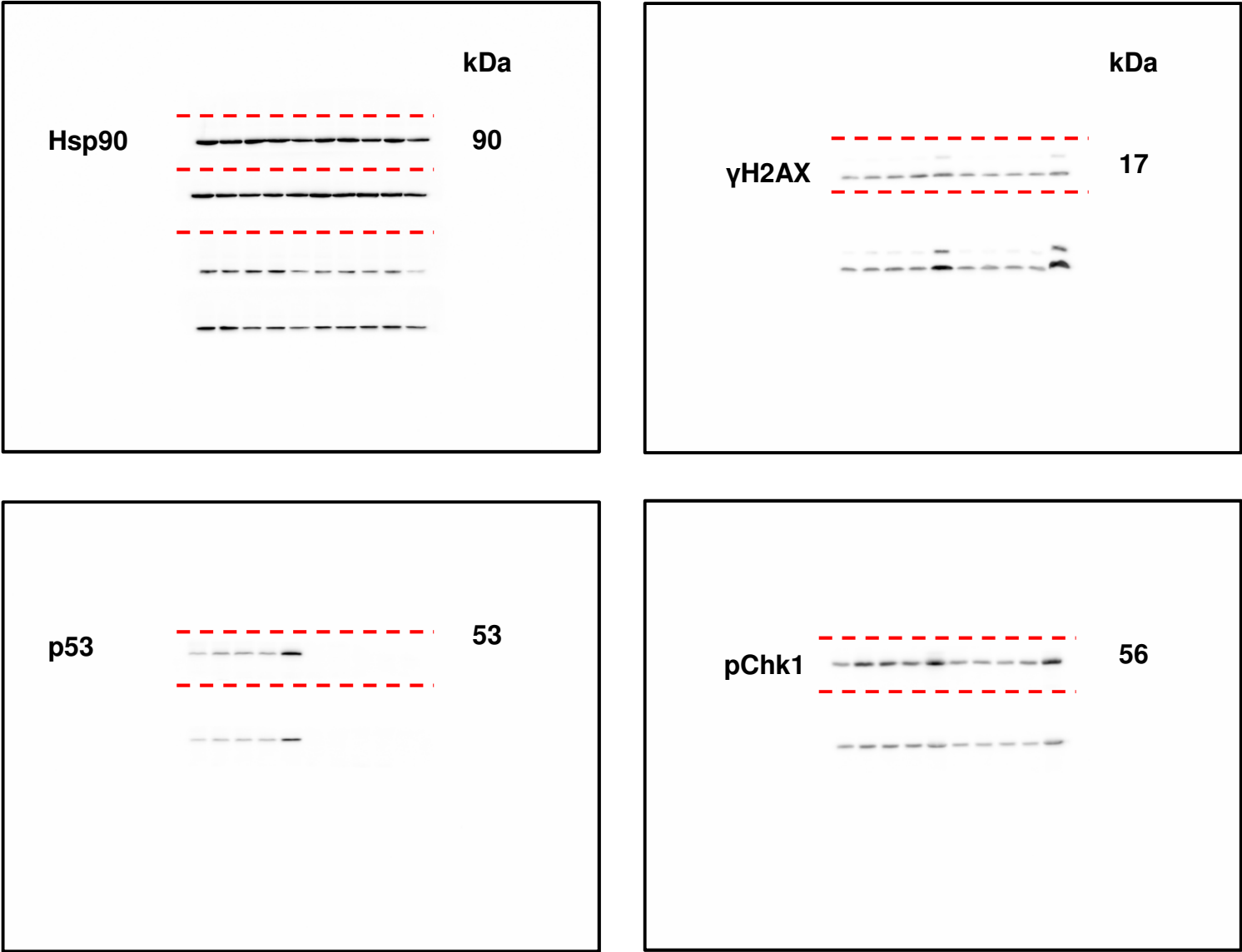

**Fig. S7D**

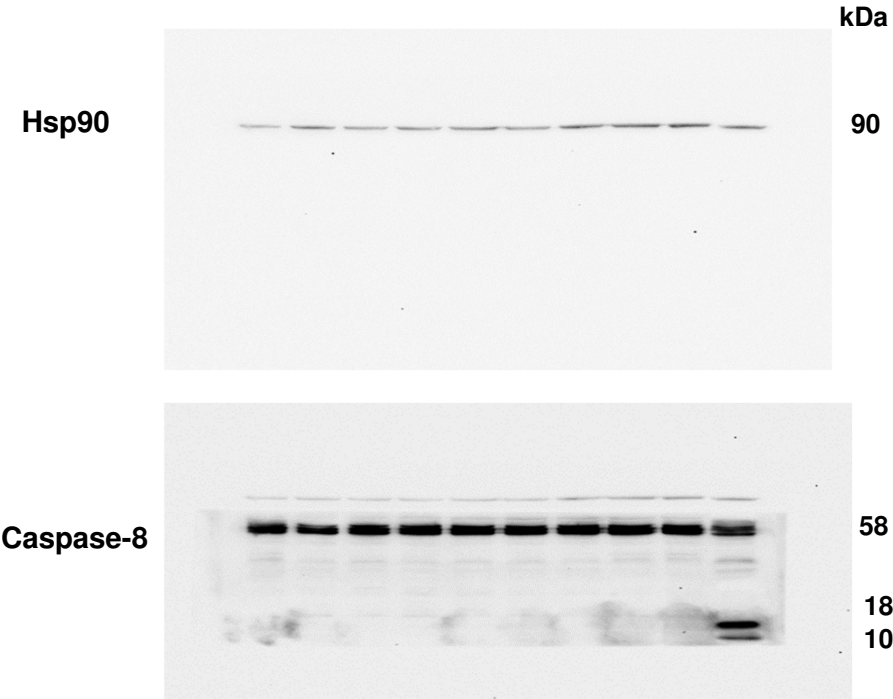

**Fig. S8A**

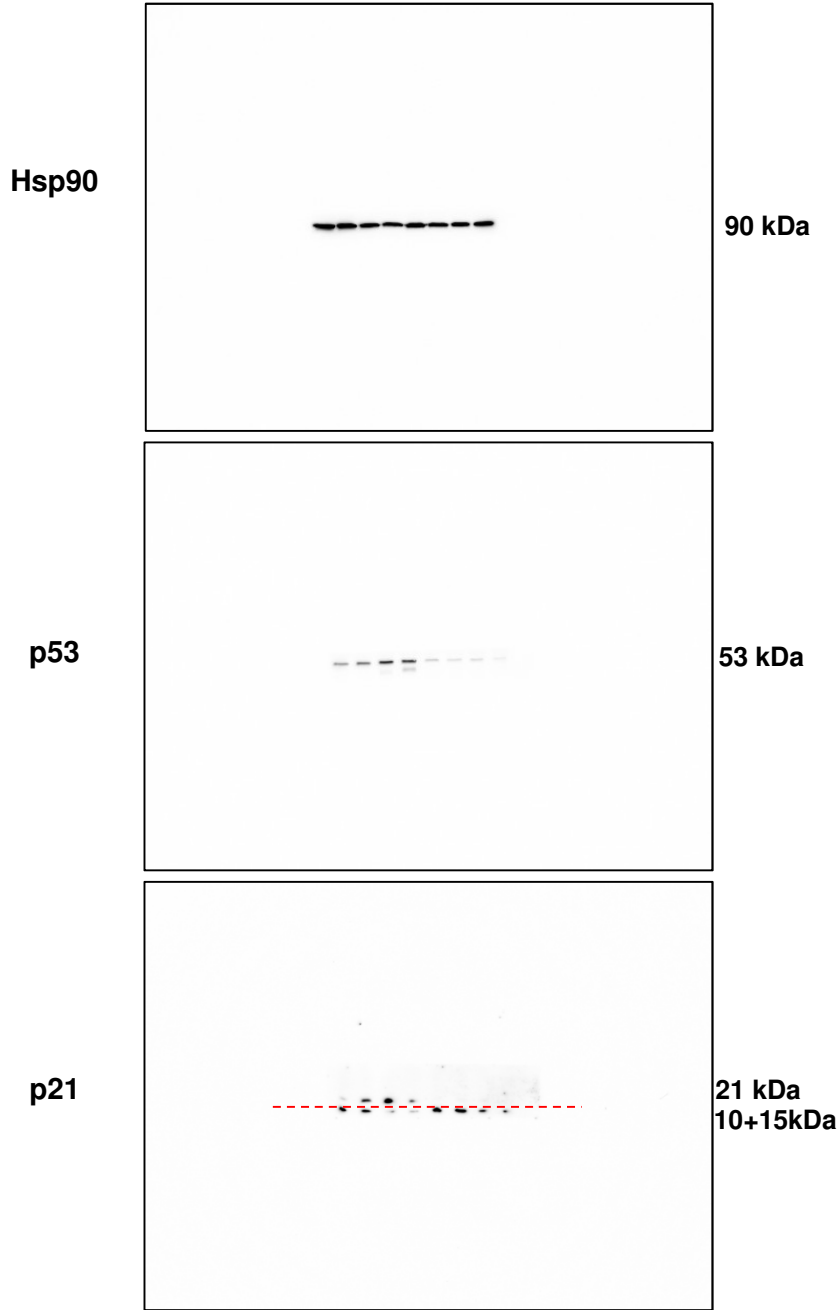

Supplement: Supplementary file 12 — original western blot data [file 41419_2022_5446_MOESM12_ESM.pdf]
