# Supplementary material for: p53 triggers mitochondrial apoptosis following DNA damage-dependent replication stress by the hepatotoxin methyleugenol
Source: Cell Death Dis. 2022 Nov 29;13(11):1009. doi: 10.1038/s41419-022-05446-9 (PMC9708695; doi:10.1038/s41419-022-05446-9)
Supplement: Supplementary file 13 — authorship contribution form [file 41419_2022_5446_MOESM13_ESM.pdf]

DECLARATION OF CONTRIBUTIONS TO ARTICLE

ADMC

Manuscript Number: CDDIS-22-1955-RR

Journal Name: *Cell Death & Disease* (the 'Journal')

Proposed Title of the Contribution:

p53 triggers mitochondrial apoptosis following DNA damage-dependent replication stress by the hepatotoxin methyleugenol (the 'Contribution)

Author(s):

Max J. Carlsson, Anastasia S. Vollmer, Philipp Demuth, Daniel Heylmann, Diana Reich, Caroline Quarz, Birgit Rasenberger, Teodora Nikolova, Thomas G. Hofmann, Markus Christmann, Julia A. Fuhlbrueck, Simone Stegmüller, Elke Richling, Alexander Cartus and Jörg Fahrer (the 'Authors')

For all *CDDis* articles, each person named as an author in the published version must be able to show he or she has contributed substantially to the article.

Authorship credit should be based on 1) substantial contributions to conception and design, acquisition of data, or analysis and interpretation of data; 2) drafting the article or revising it critically for important intellectual content; and 3) final approval of the version to be published. Authors should meet conditions 1, 2 and 3.

Any person who cannot be shown to have made a substantial contribution to the article cannot be listed as an author in the final version. The name of any person who is deemed to have made a minor contribution can, however, appear in the Acknowledgments section of the article.

Please complete the table below to indicate the contributions of all named authors to the manuscript.

Author Full Name:	Specification of Contribution to the Manuscript:
Max J. Carlsson	performed experiments, analyzed data, prepared figures, wrote paper
Anastasia S. Vollmer	performed experiments, analyzed data, prepared figures, edited paper
Philipp Demuth	performed experiments, analyzed data, edited paper
Daniel Heylmann and Diana Reich	performed experiments, analyzed data, edited paper
Caroline Quarz	prepared reagents, edited paper
Birgit Rasenberger	performed experiments, analyzed data, edited paper
Teodora Nikolova	provided methodology, performed experiments, analyzed data, edited paper
Thomas G. Hofmann	provided ressources, edited paper
Markus Christmann	provided methodology, analyzed data, edited paper
Julia A. Fuhlbrueck and Simone Stegmüller	performed experiments, analyzed data, edited paper
Elke Richling	provided ressources, edited paper
Alexander Cartus	provided methodology and protocols, edited paper
Jörg Fahrer	conceived study, analyzed data, prepared figures, wrote paper, aquired funding

Please complete the table below to indicate the contributions of all named authors to the figures.

Figure 1:

ASV generated the samples in panel A and B; JAF generated and analyzed the data in panel A & B; MJC generated the samples in panel C, D and E. CQ prepared reagents for experiments in panel D and E; SS generated and analyzed the data in panel D & E; BR and MC generated and analyzed the data in panel C; ASV and DH generated and analyzed the data in panel F, G, H & I. JF assembled the figure. ER, TGH and ATC provided resources for data shown in panel A - F.

Figure 2:

ASV generated and analyzed the data in panel A and B; TN generated and analyzed the data in panel C - E; TGH provided resources for data in panel C - E; JF assembled the figure.

Figure 3:

ASV generated and analyzed the data in panel A, B, E and F; MJC generated and analyzed the data in panel C, D, G and H; DR generated and analyzed the data in panel I; JF assembled the figure.

Figure 4:

BR generated the data in panel A; MC analyzed the data in panel A; MJC generated and analyzed the data in panel B, C and E. ASV generated and analyzed the data in panel D, F and G; JF assembled the figure.

Figure 5:

MJC generated and analyzed the data in panel A, B, C, F and G; PD generated and analyzed the data in panel F and G, JF assembled the figure.

Figure 6:

MJC generated the samples in panel A, generated and analyzed the data in panel B and C. BR and MR analyzed the data in panel A. JF illustrated the scheme in panel D and assembled the figure.

Signed for and on behalf of the Author(s):	Print Name:	Date:
	Prof. Dr. Jörg Fahrer	11-15-22
