# Supplementary material for: p53 triggers mitochondrial apoptosis following DNA damage-dependent replication stress by the hepatotoxin methyleugenol
Source: Cell Death Dis. 2022 Nov 29;13(11):1009. doi: 10.1038/s41419-022-05446-9 (PMC9708695; doi:10.1038/s41419-022-05446-9)
Supplement: Supplementary file 14 — change of authorship request form [file 41419_2022_5446_MOESM14_ESM.pdf]

SPRINGER NATURE

Change of authorship request form - Journals (pre-acceptance)

Important information. Please read.

- This form should be used by authors to request any change in authorship (adding/deleting authors) including changes in corresponding authors. This form should not be used for name changes. Please fully complete all sections. Use black ink and block capitals and provide each author's full name with the given name first followed by the family name.
- By signing this declaration, all authors guarantee that the order of the authors are in accordance with their scientific contribution, if applicable as different conventions apply per discipline, and that only authors have been added who made a meaningful contribution to the work.
- Please note, in author collaborations where there is formal agreement for representing the collaboration, it is sufficient for the representative or legal guarantor (usually the corresponding author) to complete and sign the Authorship Change Form on behalf of all authors, **next to the added/removed author(s)**. **(Complete Section 3, followed by Section 6.)**
  In author collaborations where there is no formal agreement for representing the collaboration and **there are more than 10 authors**, one may sign for all, provided the signer appends correspondence that attests that each of the authors have agreed to the change **and the added/removed authors sign the form**. **(Complete Section 3, followed by Section 6.)**
- Please note, we cannot investigate or mediate any authorship disputes. If you are unable to obtain agreement from all authors (including those who you wish to be removed) you must refer the matter to your institution(s) for investigation. Please inform us if you need to do this.
- If you are not able to return a fully completed form within **30 days** of the date that it was sent to the author requesting the change, we may have to withdraw your manuscript. We cannot publish manuscripts where authorship has not been agreed by all authors (including those who have been removed).
- Incomplete forms will be rejected.
- Please return/upload this form, fully completed, to the Journals Editorial Office. The Journal and/or Publisher will consider the information you have provided to decide whether to approve the proposed change in authorship. We may decide to contact your institution for more information or undertake a further investigation, if appropriate, before making a final decision.

SPRINGER NATURE

Change of authorship request form - Journals (pre-acceptance)

Section 1: Please provide the current title of manuscript

Manuscript ID no.: CDDIS-22-1955-T

Title: p53 triggers mitochondrial apoptosis following DNA damage-dependent replication stress by the hepatotoxin methyleugenol

Section 2: Please provide the previous authorship, in the order shown on the manuscript before the changes were introduced. Please indicate the corresponding author by adding (CA) behind the name.

	First name(s)	Family name	ORCID or SCOPUS id, if available
1st author	Max J.	Carlsson	
2nd author	Anastasia S.	Vollmer	
3rd author	Daniel	Heylmann	
4th author	Diana ~~Reich~~	Reich	
5th author	Birgit	Rasenberger	
6th author	Teodora	Nikolova	
7th author	Thomas G.	Hofmann	
8th author	Markus	Christmann	
9th author	Julia A.	Fuhlbrueck	
10th author	Simone	Stegmüller	

Please use an additional sheet if there are more than 10 authors.

↓

please see next page

SPRINGER NATURE Springer Nature is one of the world's leading global research, educational and professional publishers, created in May 2015 through the combination of Nature Publishing Group, Palgrave Macmillan, Macmillan Education and Springer Science+Business Media.

SPRINGER NATURE

Change of authorship request form - Journals (pre-acceptance)

Section 1: Please provide the current title of manuscript

Manuscript ID no.: CDDIS-22-1955T

Title: p53 triggers mitochondrial apoptosis following DNA damage-dependent replication stress by the hepatotoxin methyleugenol

Section 2: Please provide the previous authorship, in the order shown on the manuscript before the changes were introduced. Please indicate the corresponding author by adding (CA) behind the name.

	First name(s)	Family name	ORCID or SCOPUS id, if available
1 1st author	Elke	Richling	
1 2nd author	Alexander T.	Cartus	
1 3rd author	Jörg	Fahrer (CA)	0000-0001-5225-2718
4th author			
5th author			
6th author			
7th author			
8th author			
9th author			
10th author			

Please use an additional sheet if there are more than 10 authors.

SPRINGER NATURE Springer Nature is one of the world's leading global research, educational and professional publishers, created in May 2015 through the combination of Nature Publishing Group, Palgrave Macmillan, Macmillan Education and Springer Science+Business Media.

SPRINGER NATURE

Change of authorship request form - Journals (pre-acceptance)

Section 3: Please provide a justification for change. Please use this section to explain your reasons for changing the authorship of your manuscript, e.g. what necessitated the change in authorship? Please refer to the (journal) policy pages for more information about authorship. Please explain why omitted authors were not originally included and/or why authors were removed on the submitted manuscript.

Philipp Demuth was added as author, since he performed and analysed several experiments during the revision of this manuscript. The data was incorporated in Figure 5D and E, Figure S5 A–D, Figure S8 A and B. Caroline Quarz was added as author, since she synthesized, purified and characterized isotope labled DNA adduct standards, which were required to generate and analyze the data in Figure 1D and E.

Section 4: Proposed new authorship. Please provide your new authorship list in the order you would like it to appear on the manuscript. Please indicate the corresponding author by adding (CA) behind the name. <u>If the Corresponding Author has changed, please indicate the reason under section 3</u>.

	First name(s)	Family name (this name will appear in full on the final publication and will be searchable in various abstract and indexing databases)	Affiliated institute	E-mail address
1st author	Max J.	Carlsson	Technical University of Kaiserslautern	carlsson@chemie.uni-kl.de
2nd author	Anastasia S.	Vollmer	Justus Liebig University Giessen	anastasia.vollmer@outlook.com
3rd author	Philipp	Demuth	Technical University of Kaiserslautern	pdemuth@rhrk.uni-kl.de
4th author	Daniel	Heylmann	Justus Liebig University Giessen	Daniel.Heylmann@pharma.med.uni-giessen.de
5th author	Diana	Reich	Johannes Gutenberg University Mainz	diana-reich@hotmail.com
6th author	Caroline	Quarz	Technical University Kaiserslautern	quarz@chemie.uni-kl.de
7th author	Birgit	Rasenberger	Johannes Gutenberg University Mainz	rasebi00@uni-mainz.de
8th author	Teodora	Nikolova	Johannes Gutenberg University Mainz	nikolova@uni-mainz.de
9th author	Thomas G.	Hofmann	Johannes Gutenberg University Mainz	thomas.hofmann@uni-mainz.de
10th author	Markus	Christmann	Johannes Gutenberg University Mainz	mchristm@uni-mainz.de

Please use an additional sheet if there are more than 10 authors.

SPRINGER NATURE Springer Nature is one of the world's leading global research, educational and professional publishers, created in May 2015 through the combination of Nature Publishing Group, Palgrave Macmillan, Macmillan Education and Springer Science+Business Media.

SPRINGER NATURE

Change of authorship request form - Journals (pre-acceptance)

Section 3: Please provide a justification for change. Please use this section to explain your reasons for changing the authorship of your manuscript, e.g. what necessitated the change in authorship? Please refer to the (journal) policy pages for more information about authorship. Please explain why omitted authors were not originally included and/or why authors were removed on the submitted manuscript.

see last page!

Section 4: Proposed new authorship. Please provide your new authorship list in the order you would like it to appear on the manuscript. Please indicate the corresponding author by adding (CA) behind the name. If the Corresponding Author has changed, please indicate the reason under section 3.

	First name(s)	Family name (this name will appear in full on the final publication and will be searchable in various abstract and indexing databases)	Affiliated institute	E-mail address
1 1st author	Julia A.	Fuhlbrueck	Technical University of Kaiserslautern	jfuhlbrueck@gmail.com
1 2nd author	Simone	Stegmüller	Technical University of Kaiserslautern	stegmueller@chemie.uni-kl.de
1 3rd author	Elke	Richling	Technical University of Kaiserslautern	richling@chemie.uni-kl.de
1 4th author	Alexander T.	Cartus	Technical University of Kaiserslautern	a.cartus@chemservice-group.com
1 5th author	Jörg	Fahrer (CA)	Technical University of Kaiserslautern	fahrer@chemie.uni-kl.de
6th author				
7th author				
8th author				
9th author				
10th author				

Please use an additional sheet if there are more than 10 authors.

SPRINGER NATURE Springer Nature is one of the world's leading global research, educational and professional publishers, created in May 2015 through the combination of Nature Publishing Group, Palgrave Macmillan, Macmillan Education and Springer Science+Business Media.

Change of authorship request form - Journals (pre-acceptance)

Section 5: Author contribution, Acknowledgement and Disclosures. Please use this section to provide a new disclosure statement and, if appropriate, acknowledge any contributors who have been removed as authors and ensure you state what contribution any new authors made (if applicable per the journal or book (series) policy). Please ensure these are updated in your manuscript - after approval of the change(s) - as our production department will not transfer the information in this form to your manuscript.

New acknowledgements:

not applicable.

New Disclosures (financial and non-financial interests, funding):

All authors declare no conflict of interest

New Author Contributions statement (if applicable per the journal policy):

Philipp Demuth: Formal analysis, Investigation, Validation, Writing - Review and Editing
Caroline Quarz: Methodology, Resources, Writing - Review and Editing
(please see also author contribution section in the manuscript)

State 'Not applicable' if there are no new authors.

SPRINGER NATURE Springer Nature is one of the world's leading global research, educational and professional publishers, created in May 2015 through the combination of Nature Publishing Group, Palgrave Macmillan, Macmillan Education and Springer Science+Business Media.

SPRINGER NATURE

Change of authorship request form - Journals (pre-acceptance)

Section 6: Declaration of agreement. *All* authors, unchanged, new and removed *must* sign this declaration.
(NB: Please print the form, (docu)-sign and return/upload a scanned copy. Please note that signatures that have been inserted as an image file are acceptable as long as it is handwritten. Typed names in the signature box are unacceptable.) *** Please delete as appropriate. Delete all of the bold if you were on the original authorship list and are remaining as an author.**

	First name	Family name		Signature	Date
1st author	Max	Carlsson	I agree to the proposed new authorship shown in section 4 /~~**and the addition/removal*of my name to the authorship list**~~ ~~/and the proposed change in corresponding author~~		
2nd author	Anastasia	Vollmer	I agree to the proposed new authorship shown in section 4 /~~**and the addition/removal*of my name to the authorship list**~~ ~~/and the proposed change in corresponding author~~		
3rd author	Philipp	Demuth	I agree to the proposed new authorship shown in section 4 /**and the addition/~~removal~~*of my name to the authorship list** /~~and the proposed change in corresponding author~~		
4th authors	David	Heymann	I agree to the proposed new authorship shown in section 4 /~~**and the addition/removal*of my name to the authorship list**~~ ~~/and the proposed change in corresponding author~~		
5th author	Diana	Reich	I agree to the proposed new authorship shown in section 4 /~~**and the addition/removal*of my name to the authorship list**~~ ~~/and the proposed change in corresponding author~~		
6th author	Carolin	Quarz	I agree to the proposed new authorship shown in section 4 /**and the addition/~~removal~~*of my name to the authorship list** /~~and the proposed change in corresponding author~~		
7th author	Birgit	Rosenberger	I agree to the proposed new authorship shown in section 4 /~~**and the addition/removal*of my name to the authorship list**~~ ~~/and the proposed change in corresponding author~~		

SPRINGER NATURE Springer Nature is one of the world's leading global research, educational and professional publishers, created in May 2015 through the combination of Nature Publishing Group, Palgrave Macmillan, Macmillan Education and Springer Science+Business Media.

Change of authorship request form - Journals (pre-acceptance)

	First name	Family name		Signature	Date
8th author	Teodora	Nikolova	I agree to the proposed new authorship shown in section 4 /**and the addition/removal*of my name to the authorship list** /and the proposed change in corresponding author		
9th author	Thomas	Hofmann	I agree to the proposed new authorship shown in section 4 /**and the addition/removal*of my name to the authorship list** /and the proposed change in corresponding author		
10th author	Markus	Christmann	I agree to the proposed new authorship shown in section 4 /**and the addition/removal*of my name to the authorship list** /and the proposed change in corresponding author		

Please use an additional sheet if there are more than 10 authors.

↳ please see next page

In case of author collaborations with formal agreement:

	Name of consortium/consortia	First name	Family name		Signature	Date
Representative/legal guarantor				I agree to the proposed new authorship shown in section 4 /**and the addition/removal*of my name to the authorship list** /and the proposed change in corresponding author		

Both added/removed authors should complete the information in the first table under Section 6.

---- End of form ----

SPRINGER NATURE Springer Nature is one of the world's leading global research, educational and professional publishers, created in May 2015 through the combination of Nature Publishing Group, Palgrave Macmillan, Macmillan Education and Springer Science+Business Media.

SPRINGER NATURE

Change of authorship request form - Journals (pre-acceptance)

Section 6: Declaration of agreement. *All* authors, unchanged, new and removed *must* sign this declaration.
(NB: Please print the form, (docu)-sign and return/upload a scanned copy. Please note that signatures that have been inserted as an image file are acceptable as long as it is handwritten. Typed names in the signature box are unacceptable.) *** Please delete as appropriate. Delete all of the bold if you were on the original authorship list and are remaining as an author.**

	First name	Family name		Signature	Date
1st author	Julia	Fuhlbrück	I agree to the proposed new authorship shown in section 4 /~~**and the addition/removal*of my name to the authorship list** /and the proposed change in corresponding author~~		
2nd author	Simone	Stegmüller	I agree to the proposed new authorship shown in section 4 /~~**and the addition/removal*of my name to the authorship list** /and the proposed change in corresponding author~~		
3rd author	Elke	Richling	I agree to the proposed new authorship shown in section 4 /~~**and the addition/removal*of my name to the authorship list** /and the proposed change in corresponding author~~		
4th authors	Alexander	Cartus	I agree to the proposed new authorship shown in section 4 /~~**and the addition/removal*of my name to the authorship list** /and the proposed change in corresponding author~~		
5th author	Jörg	Fahrer	I agree to the proposed new authorship shown in section 4 /~~**and the addition/removal*of my name to the authorship list** /and the proposed change in corresponding author~~	Jörg Fahrer	11.10.2022
6th author			I agree to the proposed new authorship shown in section 4 /**and the addition/removal*of my name to the authorship list** /and the proposed change in corresponding author		
7th author			I agree to the proposed new authorship shown in section 4 /**and the addition/removal*of my name to the authorship list** /and the proposed change in corresponding author		

Change of authorship request form - Journals (pre-acceptance)

Section 6: Declaration of agreement. *All* authors, unchanged, new and removed *must* sign this declaration.
(NB: Please print the form, (docu)-sign and return/upload a scanned copy. Please note that signatures that have been inserted as an image file are acceptable as long as it is handwritten. Typed names in the signature box are unacceptable.) *** Please delete as appropriate. Delete all of the bold if you were on the original authorship list and are remaining as an author.**

	First name	Family name		Signature	Date
1st author	Max	Carlsson	I agree to the proposed new authorship shown in section 4 ~~**/and the addition/removal*of my name to the authorship list** /and the proposed change in corresponding author~~		13.10.22
2nd author	Anastasia	Vollmer	I agree to the proposed new authorship shown in section 4 ~~**/and the addition/removal*of my name to the authorship list** /and the proposed change in corresponding author~~		
3rd author	Philipp	Demuth	I agree to the proposed new authorship shown in section 4 **/and the addition/~~removal~~*of my name to the authorship list** ~~/and the proposed change in corresponding author~~		
4th authors	Daniel	Heymann	I agree to the proposed new authorship shown in section 4 ~~**/and the addition/removal*of my name to the authorship list** /and the proposed change in corresponding author~~		
5th author	Diana	Reich	I agree to the proposed new authorship shown in section 4 ~~**/and the addition/removal*of my name to the authorship list** /and the proposed change in corresponding author~~		
6th author	Carolin	Quarz	I agree to the proposed new authorship shown in section 4 **/and the addition/~~removal~~*of my name to the authorship list** ~~/and the proposed change in corresponding author~~		
7th author	Birgit	Rasenberger	I agree to the proposed new authorship shown in section 4 ~~**/and the addition/removal*of my name to the authorship list** /and the proposed change in corresponding author~~		

SPRINGER NATURE

Change of authorship request form - Journals (pre-acceptance)

Section 6: Declaration of agreement. ***All*** **authors, unchanged, new and removed** ***must*** **sign this declaration.**
(NB: Please print the form, (docu)-sign and return/upload a scanned copy. Please note that signatures that have been inserted as an image file are acceptable as long as it is handwritten. Typed names in the signature box are unacceptable.) *** Please delete as appropriate. Delete all of the bold if you were on the original authorship list and are remaining as an author.**

	First name	Family name		Signature	Date
1st author	Max	Carlsson	I agree to the proposed new authorship shown in section 4 /~~**and the addition/removal*of my name to the authorship list**~~ /~~and the proposed change in corresponding author~~		
2nd author	Anastasia	Vollmer	I agree to the proposed new authorship shown in section 4 /~~**and the addition/removal*of my name to the authorship list**~~ /~~and the proposed change in corresponding author~~	Vollmer	12.10.22
3rd author	Philipp	Demuth	I agree to the proposed new authorship shown in section 4 /**and the addition/~~removal~~*of my name to the authorship list** /~~and the proposed change in corresponding author~~		
4th authors	David	Heylmann	I agree to the proposed new authorship shown in section 4 /~~**and the addition/removal*of my name to the authorship list**~~ /~~and the proposed change in corresponding author~~		
5th author	Diana	Reich	I agree to the proposed new authorship shown in section 4 /~~**and the addition/removal*of my name to the authorship list**~~ /~~and the proposed change in corresponding author~~		
6th author	Carolin	Quarz	I agree to the proposed new authorship shown in section 4 /**and the addition/~~removal~~*of my name to the authorship list** /~~and the proposed change in corresponding author~~		
7th author	Birgit	Rosenberger	I agree to the proposed new authorship shown in section 4 /~~**and the addition/removal*of my name to the authorship list**~~ /~~and the proposed change in corresponding author~~		

SPRINGER NATURE Springer Nature is one of the world's leading global research, educational and professional publishers, created in May 2015 through the combination of Nature Publishing Group, Palgrave Macmillan, Macmillan Education and Springer Science+Business Media.

SPRINGER NATURE

Change of authorship request form - Journals (pre-acceptance)

Section 6: Declaration of agreement. *All* authors, unchanged, new and removed *must* sign this declaration.
(NB: Please print the form, (docu)-sign and return/upload a scanned copy. Please note that signatures that have been inserted as an image file are acceptable as long as it is handwritten. Typed names in the signature box are unacceptable.) *** Please delete as appropriate. Delete all of the bold if you were on the original authorship list and are remaining as an author.**

	First name	Family name		Signature	Date
1st author	Max	Carlsson	I agree to the proposed new authorship shown in section 4 /~~**and the addition/removal*of my name to the authorship list**~~ /~~and the proposed change in corresponding author~~		
2nd author	Anastasia	Vollmer	I agree to the proposed new authorship shown in section 4 /~~**and the addition/removal*of my name to the authorship list**~~ /~~and the proposed change in corresponding author~~		
3rd author	Philipp	Demuth	I agree to the proposed new authorship shown in section 4 /**and the addition/~~removal~~*of my name to the authorship list** /~~and the proposed change in corresponding author~~	[illegible]	13.10.22
4th authors	Daniel	Heylmann	I agree to the proposed new authorship shown in section 4 /~~**and the addition/removal*of my name to the authorship list**~~ /~~and the proposed change in corresponding author~~		
5th author	Diana	Reich	I agree to the proposed new authorship shown in section 4 /~~**and the addition/removal*of my name to the authorship list**~~ /~~and the proposed change in corresponding author~~		
6th author	Carolin	Quarz	I agree to the proposed new authorship shown in section 4 /**and the addition/~~removal~~*of my name to the authorship list** /~~and the proposed change in corresponding author~~		
7th author	Birgit	Rosenberger	I agree to the proposed new authorship shown in section 4 /~~**and the addition/removal*of my name to the authorship list**~~ /~~and the proposed change in corresponding author~~		

SPRINGER NATURE

Change of authorship request form - Journals (pre-acceptance)

Section 6: Declaration of agreement. *All* authors, unchanged, new and removed *must* sign this declaration.
(NB: Please print the form, (docu)-sign and return/upload a scanned copy. Please note that signatures that have been inserted as an image file are acceptable as long as it is handwritten. Typed names in the signature box are unacceptable.) *** Please delete as appropriate. Delete all of the bold if you were on the original authorship list and are remaining as an author.**

	First name	Family name		Signature	Date
1st author	Max	Carlsson	I agree to the proposed new authorship shown in section 4 /~~**and the addition/removal*of my name to the authorship list**~~ /~~and the proposed change in corresponding author~~		
2nd author	Anastasia	Vollmer	I agree to the proposed new authorship shown in section 4 /~~**and the addition/removal*of my name to the authorship list**~~ /~~and the proposed change in corresponding author~~		
3rd author	Philipp	Demuth	I agree to the proposed new authorship shown in section 4 /**and the addition/~~removal~~*of my name to the authorship list** /~~and the proposed change in corresponding author~~		
4th authors	David	Heylmann	I agree to the proposed new authorship shown in section 4 /~~**and the addition/removal*of my name to the authorship list**~~ /~~and the proposed change in corresponding author~~	D. Hey[illegible]	13.10.2022
5th author	Diana	Reich	I agree to the proposed new authorship shown in section 4 /~~**and the addition/removal*of my name to the authorship list**~~ /~~and the proposed change in corresponding author~~		
6th author	Carolin	Quarz	I agree to the proposed new authorship shown in section 4 /**and the addition/~~removal~~*of my name to the authorship list** /~~and the proposed change in corresponding author~~		
7th author	Birgit	Rosenberger	I agree to the proposed new authorship shown in section 4 /~~**and the addition/removal*of my name to the authorship list**~~ /~~and the proposed change in corresponding author~~		

SPRINGER NATURE Springer Nature is one of the world's leading global research, educational and professional publishers, created in May 2015 through the combination of Nature Publishing Group, Palgrave Macmillan, Macmillan Education and Springer Science+Business Media.

SPRINGER NATURE

Change of authorship request form - Journals (pre-acceptance)

Section 6: Declaration of agreement. *All* authors, unchanged, new and removed *must* sign this declaration.
(NB: Please print the form, (docu)-sign and return/upload a scanned copy. Please note that signatures that have been inserted as an image file are acceptable as long as it is handwritten. Typed names in the signature box are unacceptable.) *** Please delete as appropriate. Delete all of the bold if you were on the original authorship list and are remaining as an author.**

	First name	Family name		Signature	Date
1st author	Max	Carlsson	I agree to the proposed new authorship shown in section 4 /~~**and the addition/removal*of my name to the authorship list**~~ /~~and the proposed change in corresponding author~~		
2nd author	Anastasia	Vollmer	I agree to the proposed new authorship shown in section 4 /~~**and the addition/removal*of my name to the authorship list**~~ /~~and the proposed change in corresponding author~~		
3rd author	Philipp	Demuth	I agree to the proposed new authorship shown in section 4 /**and the addition/~~removal~~*of my name to the authorship list** /~~and the proposed change in corresponding author~~		
4th authors	Daniel	Heymann	I agree to the proposed new authorship shown in section 4 /~~**and the addition/removal*of my name to the authorship list**~~ /~~and the proposed change in corresponding author~~		
5th author	Diana	Reich	I agree to the proposed new authorship shown in section 4 /~~**and the addition/removal*of my name to the authorship list**~~ /~~and the proposed change in corresponding author~~	D. Reich	13th Oct 2022
6th author	Carolin	Quarz	I agree to the proposed new authorship shown in section 4 /**and the addition/~~removal~~*of my name to the authorship list** /~~and the proposed change in corresponding author~~		
7th author	Birgit	Rosenberger	I agree to the proposed new authorship shown in section 4 /~~**and the addition/removal*of my name to the authorship list**~~ /~~and the proposed change in corresponding author~~		

SPRINGER NATURE

Change of authorship request form - Journals (pre-acceptance)

Section 6: Declaration of agreement. *All* authors, unchanged, new and removed *must* sign this declaration.
(NB: Please print the form, (docu)-sign and return/upload a scanned copy. Please note that signatures that have been inserted as an image file are acceptable as long as it is handwritten. Typed names in the signature box are unacceptable.) *** Please delete as appropriate. Delete all of the bold if you were on the original authorship list and are remaining as an author.**

	First name	Family name		Signature	Date
1st author	Max	Carlsson	I agree to the proposed new authorship shown in section 4 **/and the addition/removal*of my name to the authorship list** /and the proposed change in corresponding author		
2nd author	Anastasia	Vollmer	I agree to the proposed new authorship shown in section 4 **/and the addition/removal*of my name to the authorship list** /and the proposed change in corresponding author		
3rd author	Philipp	Demuth	I agree to the proposed new authorship shown in section 4 **/and the addition/removal*of my name to the authorship list** /and the proposed change in corresponding author		
4th authors	David	Heymann	I agree to the proposed new authorship shown in section 4 **/and the addition/removal*of my name to the authorship list** /and the proposed change in corresponding author		
5th author	Diana	Reich	I agree to the proposed new authorship shown in section 4 **/and the addition/removal*of my name to the authorship list** /and the proposed change in corresponding author		
6th author	Carolin	Quarz	I agree to the proposed new authorship shown in section 4 **/and the addition/~~removal~~*of my name to the authorship list** /~~and the proposed change in corresponding author~~	C. Quarz	12.10.22
7th author	Birgit	Rosenberger	I agree to the proposed new authorship shown in section 4 **/and the addition/removal*of my name to the authorship list** /and the proposed change in corresponding author		

SPRINGER NATURE Springer Nature is one of the world's leading global research, educational and professional publishers, created in May 2015 through the combination of Nature Publishing Group, Palgrave Macmillan, Macmillan Education and Springer Science+Business Media.

Change of authorship request form - Journals (pre-acceptance)

Section 6: Declaration of agreement. *All* authors, unchanged, new and removed *must* sign this declaration.
(NB: Please print the form, (docu)-sign and return/upload a scanned copy. Please note that signatures that have been inserted as an image file are acceptable as long as it is handwritten. Typed names in the signature box are unacceptable.) *** Please delete as appropriate. Delete all of the bold if you were on the original authorship list and are remaining as an author.**

	First name	Family name		Signature	Date
1st author	Max	Carlsson	I agree to the proposed new authorship shown in section 4 /~~**and the addition/removal*of my name to the authorship list**~~ /~~and the proposed change in corresponding author~~		
2nd author	Anastasia	Vollmer	I agree to the proposed new authorship shown in section 4 /~~**and the addition/removal*of my name to the authorship list**~~ /~~and the proposed change in corresponding author~~		
3rd author	Philipp	Demuth	I agree to the proposed new authorship shown in section 4 /**and the addition/~~removal~~*of my name to the authorship list** /~~and the proposed change in corresponding author~~		
4th authors	Daniel	Heylmann	I agree to the proposed new authorship shown in section 4 /~~**and the addition/removal*of my name to the authorship list**~~ /~~and the proposed change in corresponding author~~		
5th author	Diana	Reich	I agree to the proposed new authorship shown in section 4 /~~**and the addition/removal*of my name to the authorship list**~~ /~~and the proposed change in corresponding author~~		
6th author	Carolin	Quarz	I agree to the proposed new authorship shown in section 4 /**and the addition/~~removal~~*of my name to the authorship list** /~~and the proposed change in corresponding author~~		
7th author	Birgit	Rosenberger	I agree to the proposed new authorship shown in section 4 /~~**and the addition/removal*of my name to the authorship list**~~ /~~and the proposed change in corresponding author~~	B. Rosenberger	14.10.22

SPRINGER NATURE Springer Nature is one of the world's leading global research, educational and professional publishers, created in May 2015 through the combination of Nature Publishing Group, Palgrave Macmillan, Macmillan Education and Springer Science+Business Media.

Change of authorship request form - Journals (pre-acceptance)

	First name	Family name		Signature	Date
8th author	Teodora	Nikolova	I agree to the proposed new authorship shown in section 4 ~~/and the addition/removal*of my name to the authorship list /and the proposed change in corresponding author~~		
9th author	Thomas	Hofmann	I agree to the proposed new authorship shown in section 4 ~~/and the addition/removal*of my name to the authorship list /and the proposed change in corresponding author~~		
10th author	Markus	Christmann	I agree to the proposed new authorship shown in section 4 ~~/and the addition/removal*of my name to the authorship list /and the proposed change in corresponding author~~	Markus Christmann	14.10.22

Please use an additional sheet if there are more than 10 authors.

↳ please see next page

In case of author collaborations with formal agreement:

	Name of consortium/consortia	First name	Family name		Signature	Date
Representative/legal guarantor				I agree to the proposed new authorship shown in section 4 **/and the addition/removal*of my name to the authorship list** /and the proposed change in corresponding author		

Both added/removed authors should complete the information in the first table under Section 6.

---- End of form ----

SPRINGER NATURE Springer Nature is one of the world's leading global research, educational and professional publishers, created in May 2015 through the combination of Nature Publishing Group, Palgrave Macmillan, Macmillan Education and Springer Science+Business Media.

SPRINGER NATURE Change of authorship request form - Journals (pre-acceptance)

	First name	Family name		Signature	Date
8th author	Teodora	Nikolova	I agree to the proposed new authorship shown in section 4 /~~**and the addition/removal*of my name to the authorship list**~~ /~~and the proposed change in corresponding author~~	[illegible] Nikolova	
9th author	Thomas	Hofmann	I agree to the proposed new authorship shown in section 4 /~~**and the addition/removal*of my name to the authorship list**~~ /~~and the proposed change in corresponding author~~		
10th author	Markus	Christmann	I agree to the proposed new authorship shown in section 4 /~~**and the addition/removal*of my name to the authorship list**~~ /~~and the proposed change in corresponding author~~		

Please use an additional sheet if there are more than 10 authors.

↳ please see next page

In case of author collaborations with formal agreement:

	Name of consortium/consortia	First name	Family name		Signature	Date
Representative/legal guarantor				I agree to the proposed new authorship shown in section 4 /**and the addition/removal*of my name to the authorship list** /and the proposed change in corresponding author		

Both added/removed authors should complete the information in the first table under Section 6.

---- End of form ----

SPRINGER NATURE Springer Nature is one of the world's leading global research, educational and professional publishers, created in May 2015 through the combination of Nature Publishing Group, Palgrave Macmillan, Macmillan Education and Springer Science+Business Media.

Change of authorship request form - Journals (pre-acceptance)

	First name	Family name		Signature	Date
8th author	Teodora	Nikolova	I agree to the proposed new authorship shown in section 4 /~~**and the addition/removal*of my name to the authorship list**~~ /and the proposed ~~change in corresponding author~~		
9th author	Thomas	Hofmann	I agree to the proposed new authorship shown in section 4 /~~**and the addition/removal*of my name to the authorship list**~~ /~~and the proposed change in corresponding author~~		13.10.22
10th author	Markus	Christmann	I agree to the proposed new authorship shown in section 4 /~~**and the addition/removal*of my name to the authorship list**~~ /~~and the proposed change in corresponding author~~		

Please use an additional sheet if there are more than 10 authors.

↳ please see next page

In case of author collaborations with formal agreement:

	Name of consortium/consortia	First name	Family name		Signature	Date
Representative/legal guarantor				I agree to the proposed new authorship shown in section 4 /**and the addition/removal*of my name to the authorship list** /and the proposed change in corresponding author		

Both added/removed authors should complete the information in the first table under Section 6.

---- End of form ----

SPRINGER NATURE Springer Nature is one of the world's leading global research, educational and professional publishers, created in May 2015 through the combination of Nature Publishing Group, Palgrave Macmillan, Macmillan Education and Springer Science+Business Media.

SPRINGER NATURE   Change of authorship request form - Journals (pre-acceptance)

Section 6: Declaration of agreement. ***All*** **authors, unchanged, new and removed** ***must*** **sign this declaration.**
(NB: Please print the form, (docu)-sign and return/upload a scanned copy. Please note that signatures that have been inserted as an image file are acceptable as long as it is handwritten. Typed names in the signature box are unacceptable.) *** Please delete as appropriate. Delete all of the bold if you were on the original authorship list and are remaining as an author.**

	First name	Family name		Signature	Date
1st author	Julia	Fuhlbrück	I agree to the proposed new authorship shown in section 4 /~~and the addition/removal*of my name to the authorship list~~ /~~and the proposed change in corresponding author~~		
2nd author	Simone	Stegmüller	I agree to the proposed new authorship shown in section 4 /~~and the addition/removal*of my name to the authorship list~~ /~~and the proposed change in corresponding author~~	[illegible]	13.10.22
3rd author	Elke	Richling	I agree to the proposed new authorship shown in section 4 /~~and the addition/removal*of my name to the authorship list~~ /~~and the proposed change in corresponding author~~	[illegible]	13.10.22
4th authors	Alexander	Cartus	I agree to the proposed new authorship shown in section 4 /~~and the addition/removal*of my name to the authorship list~~ /~~and the proposed change in corresponding author~~		
5th author	Jörg	Fahrer	I agree to the proposed new authorship shown in section 4 /~~and the addition/removal*of my name to the authorship list~~ /~~and the proposed change in corresponding author~~	Jörg Fahrer	11.10.2022
6th author			I agree to the proposed new authorship shown in section 4 **/and the addition/removal*of my name to the authorship list** /and the proposed change in corresponding author		
7th author			I agree to the proposed new authorship shown in section 4 **/and the addition/removal*of my name to the authorship list** /and the proposed change in corresponding author		

SPRINGER NATURE Springer Nature is one of the world's leading global research, educational and professional publishers, created in May 2015 through the combination of Nature Publishing Group, Palgrave Macmillan, Macmillan Education and Springer Science+Business Media.

SPRINGER NATURE

Change of authorship request form - Journals (pre-acceptance)

Section 6: Declaration of agreement. ***All*** **authors, unchanged, new and removed** ***must*** **sign this declaration.**

(NB: Please print the form, (docu)-sign and return/upload a scanned copy. Please note that signatures that have been inserted as an image file are acceptable as long as it is handwritten. Typed names in the signature box are unacceptable.) *** Please delete as appropriate. Delete all of the bold if you were on the original authorship list and are remaining as an author.**

	First name	Family name		Signature	Date
1st author	Julia	Fuhlbruck	I agree to the proposed new authorship shown in section 4 /~~**and the addition/removal*of my name to the authorship list**~~ /~~and the proposed change in corresponding author~~		
2nd author	Simone	Stegmüller	I agree to the proposed new authorship shown in section 4 /~~**and the addition/removal*of my name to the authorship list**~~ /~~and the proposed change in corresponding author~~		
3rd author	Elke	Rühling	I agree to the proposed new authorship shown in section 4 /~~**and the addition/removal*of my name to the authorship list**~~ /~~and the proposed change in corresponding author~~		
4th authors	Alexander	Cartus	I agree to the proposed new authorship shown in section 4 /~~**and the addition/removal*of my name to the authorship list**~~ /~~and the proposed change in corresponding author~~	[illegible]	12.OCT.2022
5th author	Jörg	Fahrer	I agree to the proposed new authorship shown in section 4 /~~**and the addition/removal*of my name to the authorship list**~~ /~~and the proposed change in corresponding author~~	Jörg Fahr[illegible]	11.10.2022
6th author			I agree to the proposed new authorship shown in section 4 /**and the addition/removal*of my name to the authorship list** /and the proposed change in corresponding author		
7th author			I agree to the proposed new authorship shown in section 4 /**and the addition/removal*of my name to the authorship list** /and the proposed change in corresponding author		

SPRINGER NATURE Springer Nature is one of the world's leading global research, educational and professional publishers, created in May 2015 through the combination of Nature Publishing Group, Palgrave Macmillan, Macmillan Education and Springer Science+Business Media.
